# Epigenetic Biomarkers in the Management of Ovarian Cancer: Current Prospectives

**DOI:** 10.3389/fcell.2019.00182

**Published:** 2019-09-19

**Authors:** Alka Singh, Sameer Gupta, Manisha Sachan

**Affiliations:** ^1^Department of Biotechnology, Motilal Nehru National Institute of Technology, Allahabad, India; ^2^Department of Surgical Oncology, King George Medical University, Lucknow, India

**Keywords:** biomarker, cell free DNA, diagnosis, DNA methylation, epigenetics, epithelial ovarian cancer

## Abstract

Ovarian cancer (OC) causes significant morbidity and mortality as neither detection nor screening of OC is currently feasible at an early stage. Difficulty to promptly diagnose OC in its early stage remains challenging due to non-specific symptoms in the early-stage of the disease, their presentation at an advanced stage and poor survival. Therefore, improved detection methods are urgently needed. In this article, we summarize the potential clinical utility of epigenetic signatures like DNA methylation, histone modifications, and microRNA dysregulation, which play important role in ovarian carcinogenesis and discuss its application in development of diagnostic, prognostic, and predictive biomarkers. Molecular characterization of epigenetic modification (methylation) in circulating cell free tumor DNA in body fluids offers novel, non-invasive approach for identification of potential promising cancer biomarkers, which can be performed at multiple time points and probably better reflects the prevailing molecular profile of cancer. Current status of epigenetic research in diagnosis of early OC and its management are discussed here with main focus on potential diagnostic biomarkers in tissue and body fluids. Rapid and point of care diagnostic applications of DNA methylation in liquid biopsy has been precluded as a result of cumbersome sample preparation with complicated conventional methods of isolation. New technologies which allow rapid identification of methylation signatures directly from blood will facilitate sample-to answer solutions thereby enabling next-generation point of care molecular diagnostics. To date, not a single epigenetic biomarker which could accurately detect ovarian cancer at an early stage in either tissue or body fluid has been reported. Taken together, the methodological drawbacks, heterogeneity associated with ovarian cancer and non-validation of the clinical utility of reported potential biomarkers in larger ovarian cancer populations has impeded the transition of epigenetic biomarkers from lab to clinical settings. Until addressed, clinical implementation as a diagnostic measure is a far way to go.

## KeyPoints

- Prompt diagnosis remains challenging due to non-specific symptoms in the early-stage of the disease, their presentation at an advanced stage and poor survival.
- DNA methylation occurs very early in malignant transformation and their utility as biomarker holds great promise to overcome the false positive detection of ovarian cancer using current standard serum marker CA125.
- Not even a single report has suggested or demonstrated a good epigenetic marker for early and accurate detection of OC in either tissue or fluid. Thus, early detection still remains a huge unmet need. However, analysis of a panel of aberrant methylation based epigenetic markers in blood-based non-invasive assay could pave its way into clinical implementation.

## Introduction

Ovarian cancer, a molecularly heterogeneous disease, remains the most lethal disease among gynecological malignancies. Representing as the third most frequent cancer among female gynecological system carcinoma, ovarian cancer is associated with the highest mortality rates. Despite constituting only 3% of all female cancer, the annual incidence of ovarian cancer worldwide is 220,000 with 21,290 estimated numbers of new cases and 14,600 estimated deaths annually (Siegel et al., 2015). Typical diagnosis of more than 70% of OC cases, at an advanced disease stage is one of the potent reasons for high fatality rate and carries poor prognosis with current therapies. The median age of disease presentation in ovarian cancer is 60 years and its lifetime risk is one in seventy with an overall lifetime mortality of one in ninety five (Cannistra, 2004; Howe et al., 2006).

Epithelial ovarian cancer (E0C) comprises 90% of all forms of OC cases and is characterized by heterogeneity at histopathological, clinical and molecular level. The exact cause for the ovarian malignancy still remains unknown. A strong familiar history either of ovarian or breast cancer has been described as important risk factors associated with OC. More than one-fifth of ovarian carcinomas (about 23%) have hereditary susceptibility and germline mutations of BRCA1 and BRCA2 tumor suppressor genes; in particular contribute to 65–85% of these cases (Ramus et al., 2007). An association of hormonal risk in postmenopausal women is suggested by over 50% of deaths. In addition, parity, pregnancy, lactation, tubal ligation, and oral contraceptive use are associated with reduced risk and have been found to be protective factors against disease development.

Rapid growth, non-specific clinical symptoms at early stage of the disease and lack of early diagnostic methods make prompt diagnosis challenging. As a result, EOC is typically diagnosed at an advanced stage (FIGO III/IV), when the tumor has spread beyond the pelvis and even unlikely to be completely removed by surgery. The long term survival rates for women with disseminated malignancies are low (10–30%). However, diagnosis of ovarian cancer at the localized stage (confinement of lesion still to the ovaries) is highly curable (over 95% 5 year survival rate; Siegel et al., 2011). To improve the overall survival of women diagnosed with EOC and to overcome the non-specific clinical manifestation of EOC, identification of molecular biomarkers of preclinical or early stage EOC tumors is critically needed. A better understanding of EOC genome portrait will help in the identification of promising biomarkers of clinical utility for early diagnosis of OC.

## Molecular Classification

The primary OC were classified into epithelial (60%), germ cell (30%), and sex-cord stromal tumors (8%), by the World Health Organization (WHO) classification and tumor morphology system (2014). A large majority of OC, almost 80–85%, are of epithelial origin. However, a small proportion accounting approximately 10% of all OC falls into germ cell and sex-cord stromal tumor categories (Devouassoux-Shisheboran and Genestie, 2015). Further on the basis of disease dissemination, the American Joint committee on Cancer/Tumor Node Metastasis (AJCC/TNM) and International Federation of Gynecology and Obstetrics (FIGO) staging systems, classified ovarian cancer into various stages. The confinement of tumors to the ovaries is represented by stage I and II whereas stage III is associated with local metastasis (usually lymph) and stage IV with distal organ metastases (Yarbro et al., 1999).

**Figure 1:**
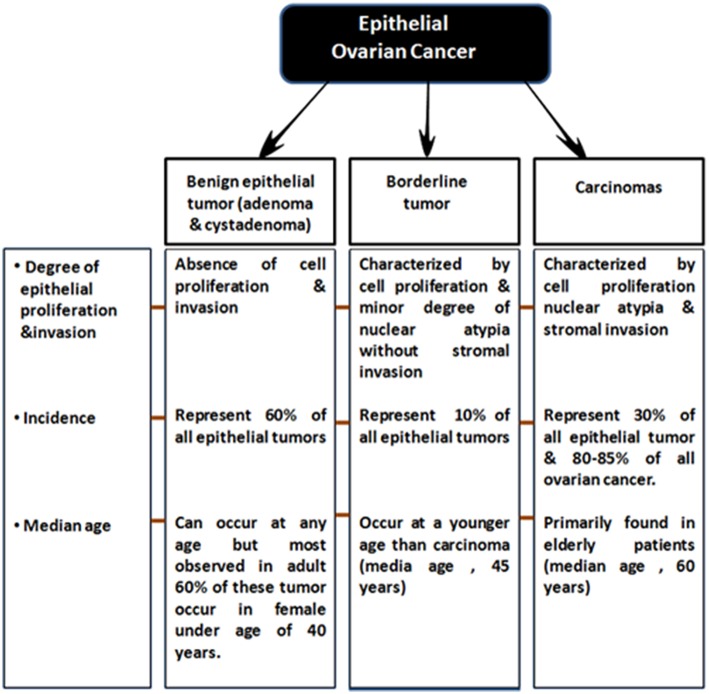
Sub-classification of epithelial ovarian tumors.

EOCs have been further sub-categorized based on following two criteria: (a) firstly, on the degree of proliferation, grade and extent of invasion into Benign (adenoma and cystadenoma), low malignant potential (LMP) and malignant (b) and secondly based on tumor histopathological grade and molecular characteristics, EOC malignant tumors are classified into serous (70%, most common), endometrioid (10–20%), clear cell (12%), mucinous (3%) and less commonly, transitional (6%), squamous, mixed, and undifferentiated (<1%) subtypes (Bowtell, 2010; Devouassoux-Shisheboran and Genestie, 2015; Earp and Cunningham, 2015; Figure 1) On the basis of histological type and grade, these tumors exhibit different genetic and epidemiological risk factors, pattern of spread, molecular abnormalities, response to targeted therapies and disease prognosis (Devouassoux-Shisheboran and Genestie, 2015; Earp and Cunningham, 2015).

**Figure 2:**
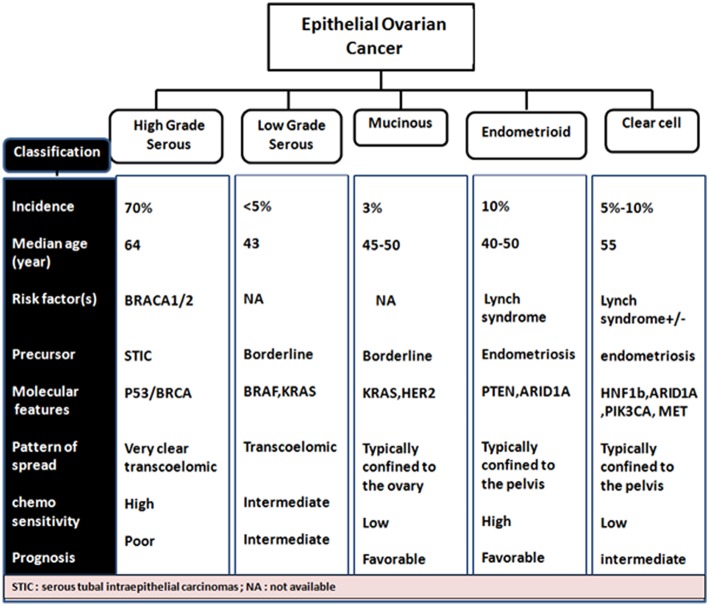
Phenotypic- genotypic classification of epithelial ovarian cancer subtypes.

Almost a decade ago, a dualistic classification system recognized Type I and Type II EOC tumors (Shih and Kurman, 2004; Vang et al., 2009). Type I EOCs are generally low grade serous carcinomas but also include mucinous, endometrioid, and clear cell subtype tumors. They are thought to arise from a low malignant potential precursor, are characterized as slow growing with low levels of chromosomal instability, intact DNA repair machinery and harbor mutations in KRAS, BRAF, and ERBB2 at a high frequency. Type II EOCs arise *de novo* and are comprised of high-grade serous carcinoma. These aggressive tumors also include malignant mixed mesodermal and undifferentiated carcinomas, are characterized by rapid growth with no identified precursor lesions, high levels of chromosomal aberrations along with high frequency of TP53, BRCA1/2 mutations. They constitute 70% of EOC cases (Jayson et al., 2014; Figure 2).

The cells of origin of ovarian cancer are still debated. Two models with respect to the origin of ovarian cancer have been proposed: (1) origin from ovarian surface epithelium (OSE), (2) from the fallopian tube. Taken together, the pro-inflammatory environment due to ovulation events, expression pattern of ovarian inclusion cysts and biomarkers which are shared by OSE and malignant growth, form the basis of first model. On contrary, tubal precursor lesions, genetic evidence of BRCA1/2 mutation carriers and recent studies strongly implicate a non-ovarian origin and form the basis of the later model. To date, neither model has evidently revealed superiority over the other. Thus, it is speculated that the HGSOC could have arisen from two different sites which undergo similar changes and could be a possible reason for tumor heterogeneity (Klotz and Wimberger, 2017). It has also been postulated that aberrantly methylated Mullerian duct cells migrate into ovarian stroma where they are supported by the epigenetically/ genetically altered stromal environment, facilitating a cascade of events which culminate in ovarian carcinogenesis. Epigenetic profiling of endocervical glandular cells would facilitate in prediction of risk or early detection of ovarian cancer (Jones et al., 2010).

## Screening and Early Detection

OC is generally characterized by few non-specific early symptoms, presentation of the disease at a late stage and poor survival. Difficulty to diagnose it in its early stages still remains challenging. Early diagnosis, screening and personalized treatment is still the biggest unmet need to combat this devastating disease. Unavailability of early cancer-specific diagnostic markers and ubiquitous acquisition of drug resistance to targeted therapies are the most striking obstacles for the effective OC treatment.

Clinically, serum antigen-125 (CA125) is the most extensively studied, established and utilized diagnostic marker of EOC, despite its elevation marked by only 47% of early-stage EOC (Woolas et al., 1993). Additionally, aberrantly elevated serum CA125 have been reported in several benign conditions of endometriosis, pregnancy, peritonitis, pelvic inflammatory disease, uterine fibroids, menstrual cycle, liver cirrhosis. Its elevation is also associated with several malignancies such as lung and colorectal cancer (Jacobs and Bast, 1989). Moreover, poor specificity, high false positive rate, and low positive prediction value make CA125 alone unsuitable as an EOC diagnostic marker. However, CA125 is more suitable markers for tumor recurrence (Clarke-Pearson, 2009).

For clinical needs to diagnose OC at an early stage, the conventional screening methods such as serum cancer antigen 125 (CA125) concentrations, transvaginal ultrasound probe and magnetic resonance imaging have not shown reliability in reducing population mortality or morbidity due to high false-negatives rates and lower sensitivity and specificity (Menon and Jacobs, 2000; Jacobs and Menon, 2004; Munkarah et al., 2007). Therefore, methods for early detection are critically required. Owing to the low incidence rate of OC amongst postmenopausal women, a logistic diagnostic screening test warrants the need of high sensitivity (>75%) and specificity (>99.6%) to attain a positive prediction value (PPV) of 10%. Novel biomarkers for early-stage diagnosis are being explored and it is more likely that a combination of biomarkers could achieve these required diagnostic criteria (Moore et al., 2010).

To determine the effect of screening on OC mortality, several randomized controlled trial in general population had been undertaken. Recently, both CA125 and transvaginal sonography (TVS) was evaluated in the Prostate, Lung, Colorectal, and Ovarian (PLCO) cancer screening trial, however no significant difference was observed in OC mortality between screening and conventional care arms (Buys et al., 2011). The United Kingdom Collaborative Trial of Ovarian Cancer Screening (UKCTOCS), being considered as the largest prospective randomized trial, comprised of over 200,000 asymptomatic postmenopausal women who were screened with TVS alone and combined TVS and CA125. Although improvement in specificity of detection was achieved on combining CA125 with TVS, however these trials failed to attain the requisite diagnostic accuracy of 99.6% specificity (Menon et al., 2009). CA125 together with HE4 has somewhat improved sensitivity and specificity of detection which correctly identified 76.4% of cancer samples and 95% of cancer negative samples. This accuracy was notably higher than either marker alone. However further validation is still required (Moore et al., 2010). According to the Guide to Clinical Preventive Services 2010–2011, it has been mentioned that neither of any screening test [serum antigen-125 (CA-125), ultrasound imaging, pelvic examination or any earlier diagnosis methods] was able to improve OC survival rates U. S. Preventive Services Task Force (2010).

The Risk of Malignancy Index, widely used at present, particularly UK, is a score based on ultrasound variables, menopausal status and CA125 (Jacobs et al., 1990). Its sensitivity is the determining criteria for a patient to be sent to experts by referring gynecologist provided objective assessment score is lower (78%) (Geomini et al., 2009). Transvaginal sonography (TVS) is based on a formal scoring model system. Though highly sensitive and being considered as an ideal method for second stage diagnosis, the major limitation associated with this method is its high dependency on individual expertise (Yazbek et al., 2008). Therefore, in clinical practice to discriminate benign and malignant ovarian tumors is still a significant challenge. The availability of biomarker or their combination which can potentially detect ovarian cancer at its earliest stage with required sensitivity and specificity would help in improving clinical outcomes.

## Markers for Ovarian Cancer Diagnosis and Management

### Protein Markers

As discussed before, a suitable screening test for OC early stage diagnosis will require high sensitivity and high specificity. Current practices for screening of OC include transvaginal ultrasonography, biomarker analysis, or a combination of both. To date, a number of potential biomarkers for early diagnosis of OC have been identified through intense research in proteomic and genomic. Here, we summarize a comprehensive account of recent researches on explored novel and robust serum based biomarkers for the non-invasive early stage screening of ovarian cancer (Table 1).

**Table 1:** Novel tumor biochemical markers for early detection of ovarian cancer.

**Biochemical marker**	**Nature**	**Origin/function**	**Status**	**Source**	**Clinical utility**	**References**
CA125	Glycoprotein antigen	Expressed by fetal amniotic epithelium and coelomic epithelium	Elevated Early stage: 47% Advanced stage: >90%	Serum	Valuable marker for tumor recurrence Limitations: Unsuitable for early detection due to insufficient sensitivity and being elevated in other conditions	Clarke-Pearson, 2009; Moore et al., 2010
HE4	Protease	Serum maturation	Elevated Serous: 93% Endometrioid: 100%	Serum	Histotype differentiation and screening	Hough et al., 2000; Lu et al., 2004; Moore et al., 2008
Mesothelin	Glycosylphosphatidylinositol- linked cell surface molecule	*Expressed by mesothelial cells *Involved in metastasis	Elevated Early stage: 60%	Urine	Early screening	McIntosh et al., 2004; Tang et al., 2013
Transthyretin	An acute phase reactant and major carrier of serum thyroxine	Tumor development	Downregulated in EOC patients	Serum	Early stage screening	Mählck and Grankvist, 1994; Schweigert and Sehouli, 2005; Nosov et al., 2009
ApoA1	Constituent of high density lipoproteins	Prevents tumor development	Downregulated in ovarian cancer patients	Serum	Early stage screening	Gadomska et al., 2005; Kim et al., 2012
Kallikrein	Family of Serine proteases *Human KLK family: 15 members *Chromosome position: 19q13.3–4	Regulates proteolytic cascades	Elevated: 12KLK/15	Serum	Elevated KLK-6 and−10 in OC cases with low level of CA125. Useful marker for OC detection	Borgoño and Diamandis, 2004; Rosen et al., 2005
Osteopontin	An adhesive glycoprotein	*Synthesized by osteoblasts and vascular endothelial cells *Associated with bone remodeling and immune function	Elevated in invasive and borderline ovarian cancer tumors	Plasma	Early stage screening	Kim et al., 2002

Although being considered as the “gold standard” biomarker for detection of OC, its clinical relevance mainly falls in evaluating disease recurrence. Other biochemical markers such as lysophosphatidic acid, human epididymis protein 4 (HE4), inhibins (which are members of TGF-β subfamily), Mesothelin (associated with migration and metastasis) (Huang et al., 2006), Osteopontin, and YKL-40 have been reported to be elevated in sera of patients with OC amongst various studies, which could be of diagnostic significance for improved cancer detection, most likely in various combination with one another and /or with CA125 (Rosenthal et al., 2006; Moore et al., 2010). The most promising molecular biomarker of all these, to date are HE4 and Mesothelin. So far, US FDA has only approved CA125 and HE4 for monitoring disease progression/recurrence, but not for screening purpose (Rosenthal et al., 2006).

For the triage of pelvic mass, the multivariate index assay OVA1, constituting measurements of 5-proteins: CA125-II, apolipoprotein A1, transthyretin, beta 2 microglobulin, and transferrin, has been approved by FDA since 2009. Although, the test had improved sensitivity but compromised in revealing diagnostic potential with its low specificity upon replacement of CA125 with the multivariate index assay (Nguyen et al., 2013). Elevated levels of Kallikrein 6 and 7 (KLK6 and KLK7) was reported in sera of ovarian carcinoma subtypes, depicting their potential to improve early detection of OC. Other biomarkers with potential clinical significance for early diagnosis in women with EOC include GSTT1, Prostasin (PRSS8), KLK6, KLK7, FOLR1, and ALDH1, which are currently under research and clinical trials (Sarojini et al., 2012).

Evaluation of several prediagnostic multimarker panels along with PLCO screening trial has identified promising biomarkers which are able to distinguish ovarian cancer cases from normal control groups; for instance, a four biomarker panel consisting of CA-125, HE4, CEA, and VCAM-1 effectively discriminated early stage OC from healthy controls with sensitivity of 86% at 98% specificity (Lin et al., 2009). Another panel constituting of CA-125, ApoA1, TTR, and H418, was able to differentiate OC patients at early stage of disease from cancer-free healthy control samples with 74% sensitivity at 97% specificity (Zhang et al., 2004). Still to date, no panel of biomarkers that has been examined amongst numerous studies could outperform CA125 alone, in distinguishing between the two groups. The sensitivity and specificity of serum based non-invasive biomarkers for improved ovarian cancer detection from various studies as well as the currently active/completed clinical trials evaluating potent biochemical markers of clinical significance for early diagnosis of EOC are summarized in Tables 2, 3 respectively.

**Table 2:** Specificity and sensitivity of early detection biomarkers for ovarian cancer from various studies.

**Biochemical biomarker**	**Source**	**Population tested**	**Clinical prediction**		**References**
			**Sensitivity**	**Specificity**	
HE4	Serum	147 Cancer (111 ovarian cancer cases), 285 Benign, 66 controls	79.3%	98.9%	Molina et al., 2011
HE4 + CA125	Serum	383 Benign and 89 Cancer	100% 92.30%	74.20% 76.0 %	Moore et al., 2011
Osteopontin	Plasma	46 Benign, 47 cancer, 51 ovarian cancer, 107 controls	-	-	Kim et al., 2002
Prostasin + CA125	Serum	64 cancer, 137 control	92%	94%	Mok et al., 2001
KLK6	Serum	141 Benign, 146 ovarian cancer, 97 controls	21–26%	95%	Diamandis et al., 2003
KLK6+ CA125	Serum	141 Benign, 146 ovarian cancer, 97 controls	42%	90%	Diamandis et al., 2003
B7-H4	Serum	1,023 cancer, 997 Benign (236 ovarian cancer cases, 260 controls)	45%	97%	Simon et al., 2006
B7-H4 + CA125	Serum	1,023 cancer, 997 Benign (236 ovarian cancer cases, 260 controls)	65%	97%	Simon et al., 2007
CA125/IL-6/IL-8/VEGF/EGF	Serum	44 Early-stage cancers 37 Benign, 45 controls	84%	95%	Gorelik, 2005
CA125/IL-6/G-CSF/VEGF/EGF	Serum	44 Early-stage cancers 37 Benign, 45 controls	86.5%	93%	Gorelik, 2005
CA125/HE4/Glycodelin/ PLAUR/MUC1/PAI-1	Serum	200 Cancers (133 stage I/II), 396 Healthy controls	80.5%	96.5%	Havrilesky et al., 2008
Leptin/Prolactin/ Osteopontin/IGF2	Serum	100 Cancers, 106 controls	95%	94%	Mor et al., 2005
CA125 HE4 Mesothelin	Serum	143 Cancers, 124 benign, 344 controls	78% 68–82% 31–44%	98% 98% 98%	Shah et al., 2009
Leptin/Prolactin/ Osteopontin/IGF2/MIF/ CA125	Serum	Training: 113 cancers, 181 controls Test: 43 cancers, 181 Controls	95.3%	99.4%	Visintin et al., 2008
CA125/ CA19- 9 /EGFR /CRP/ Myoglobin/APOA1/ APOC3/MIP1A/ IL-6/IL-18/ Tenascin C	Serum	115 Cancers, 93 benign 24 Controls, 13 non-ovarian cancers	91.3%	88.5%	Amonkar et al., 2009
CA-125, HE4, SI	Serum	74 cancer, 137 controls	84%	98.5%	Andersen et al., 2010
RIM, ROMA, CA-125, HE4,	Serum	445 Benign, 31 borderline, and 162 malignant tumors	Postmenopausal 89, 91, 92, and 72% Premenopausal 87, 87, 96, and 83%	Postmenopausal 80, 77, 80, and 92% Premenopausal 90, 81, 60, 91%	Lycke et al., 2018
CA-125 HE4 TTR	Serum	130 Benign, 126 ovarian cancer, 400 controls	64.29% 46.4% 78.6%	53.57% 43.3% 68.8%	Zheng et al., 2018
CA-125, HE4, TK1	Serum	75 ovarian cancer, 40 Benign, 35 controls	94.18%	79.53%	Xi et al., 2017
CA-125+HE4, HE4+FOLR1	Serum	150 benign controls, 216 ovarian cancer, 20 controls	67% 65%	95% 95%	Leung et al., 2016
CA-125, ApoA1, TTR	Serum	200 cancer, 142 controls	74%	97%	Zhang et al., 2004
CA125, HE4, MMP-7, CA72-4	Serum	142 stage I cancer, 217 controls	83.2%	98%	Simmons et al., 2016
CA-125, CA 72–4, MCSF	Plasma	123 cancer, 224 controls	70%	98%	Edgell et al., 2010
CA-125, TTR, ApoA1	Serum	20 cancer, 82 controls	89%	92%	Su et al., 2007
CA-125, HE4, CEA, VCAM-1	Serum	456 cancer, 2,000 controls	86–93%	98%	Lin et al., 2009
CA-125, CRP, SAA, IL-6, IL-8	Plasma	150 cancer, 212 controls	94.1%	91.3%	Edgell et al., 2010
S100A4	Serum	160 cancer, 52 Benign, 52 controls	78%	92%	Lv et al., 2018
KPNA2	Serum	162 cancer, 48 controls	71.4%	81.2%	Huang et al., 2017
Septin-9 Clusterin	Plasma	137 EOC, 12 borderline,51 benign, 58 controls	82.5% 71.5%	50.0% 41.4%	Lyu et al., 2018

**Table 3:** Clinical trials (currently active or completed) for evaluating novel biomarkers of ovarian cancer.

**Biochemical marker**	**Setting**	**Phase**	**Samples (*n*)**	**Status**	**Clinical trial no**.	**Primary clinical outcome**	**References**
All biomarkers	Adnexal mass	1	500 (E)	Completed	NCT01466049	Screening	NA
HE4 + CA125	Pelvic mass	0	566	Completed	NCT00315692	Cancer vs. benign disease	Moore et al., 2009
CA125	Low risk women	1	9500 (E)	Recruiting	NCT00539162	Rate of increase in CA125 over time	NA
HE4 + CA125	Adnexal mass	1	512	Completed	NCT00987649	Initial cancer risk assessment	NA
CA125+ HE4	High risk women	1	1208 (E)	Active, not recruiting	NCT01121640	PPV of screening protocol	NA
CA125	High risk women	2	2400 (E)	Withdrawn	NCT00080639	Screening	NA
Mesothelin	Low risk women	0	250 (E)	Unknown	NCT000155740	Screening	NA
FOLR1	Stage I ovarian cancer	2	50 (E)	Terminated	NCT01511055	Sensitivity and specificity of Intraoperative imaging (IOI) with folate	NA
CA125 + TVU	Ovarian disease	0	750 (E)	Terminated	NCT01292733	CA125 measurement in blood over time	NA
CA125± TVU	Postmenopausal	0	48230	Completed	NCT00058032	Screening post menopausal women	Menon et al., 2009; Jacobs et al., 2011
CA125	High risk women	0	2430	Unknown	NCT00039559	Sensitivity and specificity of early detection for ovarian cancer	NA
CA125+ TVU	High genetic risk women	0	5000 (E)	Completed	NCT00033488	Screening women at high genetic risk for ovarian cancer	NA
CA125	High risk women	0	6000 (E)	Recruiting	NCT00005095	Screening	NA
Combined methods	Ovarian neoplasms	0	36000	Unknown	NCT01178736	Low- cost screening	NA
Interventional	High risk women	0	1500	Recruiting	NCT00849199	Genetic testing, screening	NA
All biomarkers	High risk women	0	250 (E)	Unknown	NCT00854399	Overall survival	NA
Tumor markers	High risk women	0	5000	Completed	NCT00267072	Early stage detection	NA
DNA markers	Ovarian cancer	0	118 (E)	Active, not recruiting	NCT00879840	Assessment of screening modalities	NA
BRCA1/2 Mutation	Ovarian neoplasms	0	1500	Completed	NCT00001468	Identifying BRCA1/2 mutation	NA
BRCA	Epithelial ovarian cancer	0	600 (E)	Completed	NCT03229122	Genetic Testing of BRCA	NA
All biomarkers	High risk women	0	500(E)	Recruiting	NCT03150121	Identification of uterine lavage-based biomarkers for early detection	NA
All biomarkers	High risk women	0	6000	Recruiting	NCT00005095	Early stage detection and prevention	NA
CA125	High genetic risk women	0	40	Completed	NCT00043472	Screening	NA
DNA markers	Women with serous epithelial ovarian cancer	0	250	Not yet recruiting	NCT03622385	Early detection of high grade serous epithelial ovarian cancer	NA

*TVU, transvaginal ultrasonography; (w), women; (E), estimated enrollment; IOI, intraoperative imaging. Source: http://clinicaltrials.gov/*.

### Genetic Marker

About 23% of ovarian tumors have been associated with hereditary conditions and the genetic abnormalities in about 65–85% of hereditary ovarian carcinomas is the germline mutation in BRCA (breast cancer early onset genes BRCA1 and BRCA2) genes which are essential for DNA repair as well as in maintaining genomic stability and integrity. The cumulative lifetime risk of EOC for a woman with BRCA1 and BRCA2 mutation is 39–46% and 12–20%, respectively (Ramus et al., 2007). Lifetime risk to develop breast cancer and ovarian cancer is enhanced up to 85% and up to 54% respectively in the carriers of BRCA1 and BRCA2 mutations. Association of several tumor suppressor genes and oncogenes (tumor suppressor gene TP53 in Li- Fraumeni syndrome, mismatch repair genes (MMR) in Lynch syndrome, genes involved with double strand break repair system: BARD1, CHEK2, RAD51, and PALB2) with hereditary ovarian cancer has been reported. Till date, around 16 genes have been reported to be associated with hereditary ovarian carcinogenesis while several other mutations are yet unknown and need to be further explored (Toss et al., 2015).

### Epigenetic Marker

Epigenetics is the mechanism for the regulation of gene expression without any alternation in the primary DNA sequence (Jones and Laird, 1999; Jones and Baylin, 2002; Feinberg and Tycko, 2004). DNA methylation, modification of histone proteins and miRNAs are the key modulator in regulating several cellular processes such as cell differentiation, embryogenesis, inactivation of X chromosome, genome imprinting, and many others (Jones, 2001; Reik and Lewis, 2005; Kacem and Feil, 2009; Portela and Esteller, 2010). The epigenetic alternations involve interplay between DNA methylation, histone modification and micro RNA expression to modulate gene expression during development and cancer progression. (1) The global hypomethylation, largely of repetitive DNA which results in demethylation of several oncogenes and (2) localized hypermethylation at promoters of various tumor suppressor genes leading to their transcriptional silencing, are two opposite epigenetic phenomenon involved in tumorigenesis (Sharma et al., 2010). DNA methyltransferase (DNMT) mediated methylation of deoxycytosine located within the CpG dinucleotides is the best known and widely studied epigenetic mechanism leading to transcription repression in cancer (Bird and Wolffe, 1999; Hendrich and Bird, 2000; Bird, 2002). DNA methylation is known to be the earliest event during carcinogenesis and plays a crucial role in silencing of tumor suppressor genes (Sharma et al., 2010; Teschendorff and Widschwendter, 2012; Teschendorff et al., 2012, 2016; Bartlett et al., 2016). Promoter methylation mediated epigenetic silencing of gene is regulated by the recruitment of MBD (methyl CpG binding proteins such as MeCP2, MBD1, MBD2, and MBD4) which in turn regulates chromatin state by recruiting histone modifying and chromatin-remodeling complexes (repressors) at the site of methylation, which subsequently generates condensed chromatin structure and results in transcriptional repression (Esteller, 2007; Lopez-Serra and Esteller, 2008). On contrary, epigenetic activation of gene is regulated by recruitment of Cfp1 and histone methyltransferase Setd1 which aids in generating an open chromatin structure by creating domains which are enriched with active histone marks (acetylation and H3K4 trimethylation) (Thomson et al., 2010, p. 1; Jones and Baylin, 2007; Supplementary Figure 1). Increasing evidences has revealed the significant role of DNA methylation in cancer development and progression, right from transcriptional silencing of tumor suppressor genes to the activation of oncogenes and consequently promoting metastasis (Costello and Plass, 2001; Herman and Baylin, 2003; Wilting and Dannenberg, 2012). Apparently, it is quite evident now that DNA methylation plays an equal or possibly even greater role than the genetic lesion such as mutations, deletion and translocations which have been associated for long, with malignant transformations and carcinogenesis (Chan T. A. et al., 2008). For instance, though the familial breast cancer susceptibility gene 1 (BRCA1) mutations contributes to 5–10% of EOC, promoter hypermethylation of non-mutated BRCA1 allele is the second disruptive event to the development of this cancer (Barton et al., 2008).

#### Tissue Biomarkers

##### Diagnosis

So far, several methylation based signatures have been reported in EOC. Here, we summarize an overview of some of the extensively studied potential biomarkers of diagnostic utility in ovarian cancer (Table 6). In ovarian cancer, a large number of tumor suppressor genes have been identified to be silenced by promoter hypermethylation and downregulated includes DAPK, LOT1, TMS1/ASC, and PAR4 (pro-apoptotic function and cell cycle regulation), p16, SPARC, ANGPTL2, and CTGF (tumor suppressor activity), ICAM-1 and CDH1 (cell adhesion), PEG31 (role in imprinting) and many others (Tables 4, 5). In ovarian cancer, some of the most frequently methylated genes include OPCML (tumor suppressor activity), TES (involved in regulation of cell motility) and RASSF1A (tumor suppressor activity as well as an inhibitor of the anaphase-promoting complex) (Barton et al., 2008). Promoter methylation of HOXA10 and HOXA11, which are involved in very early ovarian tumor initiation effectively distinguished normal and malignant ovaries (Fiegl et al., 2008; Widschwendter et al., 2009). Methylation induced silencing of PTEN has also been frequently observed in primary epithelial ovarian carcinomas (Kurose et al., 2001). CTGF (encodes the connective tissue growth factor) (Kikuchi et al., 2007; Barbolina et al., 2009), CCBE1 (hypothesized to be involved in regulation of cell motility) (Barton et al., 2010), HIC1 (a p53 target gene) (Rathi et al., 2002), CDH13 (Makarla et al., 2005), and CDH1 (the loss of which correlates with the upregulation of matrix metalloproteinases and metastasis- promoting protein a 5- integrin) (Sawada et al., 2008) act as metastasis suppressors. Methylation induced repression of these suppressors correlates with invasive EOC.

**Table 4:** List of most frequently epigenetically dysregulated genes in ovarian cancer.

**S. No**	**Gene**	**Function**	**Epigenetic event**	**References**
1.	Survivin	Apoptotic inhibitor	Downregulated	Mirza et al., 2002
2.	GATA 4	Transcription factor	Downregulated	Caslini et al., 2006; Cai et al., 2009
3.	APC	Regulation of cell adhesion	Downregulated	Tam et al., 2007
4.	ARHI	Ras homolog, maternally imprinted putative tumor suppressor. Negative regulator of cancer growth and progression	Downregulated	Yu et al., 2003; Fu et al., 2015
5.	BRCA 1	DNA damage response	Downregulated	Baldwin et al., 2000; Wilcox et al., 2005; Press et al., 2008
6.	DAPK	Apoptosis	Downregulated	Bai et al., 2004
7.	Estrogen receptor β	Transcriptional activator	Downregulated	Suzuki et al., 2008
8.	hMLH 1	DNA mismatch repair	Downregulated	Meng et al., 2008; Zhang H. et al., 2008
9.	hMSH 2	DNA mismatch repair	Downregulated	Zhang H. et al., 2008
10.	ICAM 1	Cell/matrix adhesion	Downregulated	Arnold et al., 2001
11.	LOT1	Apoptosis	Downregulated	Abdollahi et al., 2003; Kamikihara et al., 2005
12.	OPCML	Cell adhesion	Downregulated	Sellar et al., 2003; Teodoridis et al., 2005; Zhang J. et al., 2006
13.	PACE-4	Serine protease	Downregulated	Fu et al., 2003
14.	RASSFIA	Microtubule stability	Downregulated	Agathanggelou et al., 2001; Yoon et al., 2001
15.	PEG 3	Apoptosis	Downregulated	Feng W. et al., 2008
16.	DLEC 1	Unknown	Downregulated	Kwong et al., 2006
17.	ARLTS 1	Premature termination of translation	Downregulated	Petrocca et al., 2006
18.	TCEAL 7	Cell death regulation	Downregulated	Chien et al., 2005
19.	P16	Cell cycle control	Downregulated	Milde-Langosch et al., 1998; Katsaros et al., 2004
20.	TMS1	Apoptosis	Downregulated	Akahira et al., 2004a
21.	WT1	Transcription factor	Downregulated	Kaneuchi et al., 2005
22.	14-3-3 SIGMA	Regulation of cell growth and differentiation	Downregulated	Kaneuchi et al., 2004
23.	DR 4	Apoptosis	Downregulated	Horak et al., 2005
24.	FBXO 32	Apoptosis (?)	Downregulated	Chou et al., 2010
25.	IGFBP-3	Antiproliferative, pro-apoptosis, and invasion suppressor	Downregulated	Torng et al., 2009
26.	SFRP5	Modulator of Wnt signaling	Downregulated	Su et al., 2010
27.	CCBE 1	Migration and cell survival	Downregulated	Barton et al., 2010
28.	RUNX3	Transcription factor	Downregulated	Zhang et al., 2009
29.	CHFR	Cell cycle control	Downregulated	Gao et al., 2009
30.	Maspin(SERPINBS)	Protease inhibitor	Expressed	Rose et al., 2006
31.	FANCF	DNA repair(?)	Up regulated	Taniguchi et al., 2003
32.	Synuclein gamma	Unknown	Up regulated	Gupta et al., 2003
33.	TUBB3	Formation of microtubules	Up regulated	Izutsu et al., 2008
34.	CLDN3	Tight junction protein	Up regulated	Honda et al., 2007
35.	HOXA10	Transcription factor	Up regulated	Cheng et al., 2010
36.	FBXW7	Ubiquitin ligase mediates ubiquitylation of oncoproteins	Downregulated	Kitade et al., 2016
37.	SPARC	Membrane-associated glycoprotein, normal development, anti-proliferative, and de-adhesive properties angiogenesis, ECM production	Downregulated	Socha et al., 2009
38.	HIC1	Transcriptional repressor	Downregulated	Pieretti et al., 1995; Rathi et al., 2002; Teodoridis et al., 2005; Tam et al., 2007
39.	Rarβ	Transcriptional regulator of cell growth	Downregulated	Makarla et al., 2005; Tam et al., 2007
40.	GSTP1	Intra cellular detoxification	Downregulated	Makarla et al., 2005; Bol et al., 2010
41.	TBX15	Development of mesodermal derivative	Downregulated	Gozzi et al., 2016
42.	HUSIF 1	Modulate heparin binding growth factor signaling promote Wnt signaling pathway	Downregulated	Staub et al., 2007
43.	SFRP1	Inhibitor of Wnt/β-catenin signaling pathway	Downregulated	Su et al., 2009; Kardum et al., 2017
44.	RunX1T1	Putative SMAD4 target/TGFβ/SMAD4 signaling	Downregulated	Yeh et al., 2011
45.	ANGPTL2	Secreted glycoprotein involved in angiogenesis	Downregulated	Kikuchi et al., 2008
46.	CTGF	Adhesion molecule, motility modulator	Downregulated	Kikuchi et al., 2007
47.	FOXD3	Transcriptional regulator of development, cell maintenance, and lineage specification	Downregulated	Luo et al., 2019
48.	NISCH	Encodes imidazoline receptor Nischarin, regulates cellular migration, and invasion upon interacting with PAK1, LIMK, Rac1, and LKB1	Downregulated	Li J. et al., 2015
49.	ABCA1	A TGF-β target, regulator of cholesterol efflux and metabolism	Downregulated	Chou et al., 2015, p. 1
50.	TIMP2	A EZH2 target, endogenous regulator of matrix metalloproteinases, repressor of metastasis	Downregulated	Yi et al., 2017
51.	PCDH17	Transmembrane protein belonging to cadherin superfamily, potential calcium-dependent cell-adhesion protein	Downregulated	Baranova et al., 2018
52.	LDOC1	A nuclear transcription factor, regulator of NFκB Signaling	Downregulated	Buchholtz et al., 2014
53.	RGS2	Regulator of GTPase activity of G protein subunits. Negative regulator of angiotensin-activated signaling pathway	Downregulated	Cacan, 2017
54.	PRTFDC1	Unknown	Downregulated	Cai et al., 2007
55.	DDR	Subclass of RTKs, associated with cell differentiation, proliferation, adhesion, migration, and invasion	Downregulated	Chung et al., 2017
56.	ARNTL	Circadian gene	Downregulated	Yeh et al., 2014
57.	GULP1	Apoptosis, lipid homeostasis, regulator of Arf6-mediated signaling	Downregulated	Maldonado et al., 2018, p. 1
58.	TGFB1	Adhesion, essential for function of microfibrils and interacts with fibronectin and integrins	Downregulated	Kang et al., 2010
59.	SALL2	Cellular quiescence factor, neural development	Downregulated	Sung et al., 2013
60.	PDZ-LIM	Ubiquitination of nuclear p65, inflammation	Downregulated	Zhao et al., 2016
61.	KLF11	Inhibitory regulator of TGFβ signaling, promotes apoptosis	Downregulated	Wang et al., 2015
62.	GBGT1	Encodes glycosyltransferase which plays role in synthesis of Forssman glycolipid	Downregulated	Jacob et al., 2014

**Table 5:** List of hypermethylated genes in ovarian cancer.

**Gene**	**Frequency of hypermethylation in ovarian cancer**	**Ovarian cancer subtype**	**Frequency of hypermethylation in normal tissue**	**Method**	**References**
RASSF1A	15.6–50%	S, M, E, CC	0–13%	MSP	Yoon et al., 2001; Makarla et al., 2005; Choi et al., 2006; Bol et al., 2010
DAPK	50–67% (full)	S, CC, E, M, CS, PDA	50% (Partial)	MSP	Collins et al., 2006; Häfner et al., 2011
p16(CDKN2A)	16.9–42%	S, M, E	0–25%	MSP	Strathdee et al., 2001; Rathi et al., 2002; Tam et al., 2007
HIC1	15.9–51.7%	Not specific	12.5–19%; 11.1%	MSP	Strathdee et al., 2001; Ongenaert et al., 2008
OPCML	46.5–83.3%	Not specific	0%	Restriction enzyme cut analysis, MSP	Czekierdowski et al., 2006a; Zhang J. et al., 2006
MLH1	10%	S, E, M, CC, MIX	0% ADJ NLS	MSP	Strathdee et al., 2001
TERT	29.8%	S, M, E, CC	30%	qMSP	Widschwendter A. et al., 2004
PTEN	16.9%	E, S, M, CC	0%	MSP	Ongenaert et al., 2008
ING1	24%	S, M, E, CC, PDA	0%	MSP	Shen et al., 2005
ITGA8	13.3%	S, E, M, CC, SCC	0% END cyst	MSP	Cai et al., 2007
MGMT	9%	S, M, CC, E, UN	16%	MSP	Makarla et al., 2005
MINT25	16%	S, E, M, CC, MIX	0% ADJ NLS	MSP	Strathdee et al., 2001
APC	18–47.2%	S, M, CC, E, UN	0–25%	MSP	Rathi et al., 2002; Makarla et al., 2005; Ongenaert et al., 2008
BRCA1	10–24%	S, E, M, CC, MIX	0–5.5% ADJ NLS	MSP	Strathdee et al., 2001; Rathi et al., 2002; Ibanez de Caceres et al., 2004
CASP8	3%	S, E, M, CC, MIX	0% ADJ NLS	MSP	Strathdee et al., 2001
CDH1 (E-cadherin)	26–29%	S, M, CC, E, UN	6%	MSP	Rathi et al., 2002; Makarla et al., 2005
CDH13 (H-cadherin)	18–22%	S,M, CC, E, UN	8–13%	MSP	Rathi et al., 2002; Makarla et al., 2005
DCR1	43%	NS	0%	MSP	Shivapurkar et al., 2004
GPR150	26.6%	S, E, M, CC, SCC	0% END cyst	MSP	Cai et al., 2007
Htr (TERC)	24%	S, E, M, CC, MIX	0% ADJ NLS	MSP	Strathdee et al., 2001

*ADJ NLS, Adjacent normals; CC, Clear cell; CS, Carcinosarcoma; E, Endometroid; END, Endometrial; M, Mucinous; MIX, Mixed; MSP, Methylation-specific PCR; NS, Not specified; PDA, Poorly differentiated adenocarcinoma; QMSP, Quantitative methylation-specific PCR; S, Serous; SCC, Squamous cell carcinoma; UN, Undifferentiated*.

**Table 6:** Epigenetic biomarkers for ovarian cancer detection.

**Epigenetic marker**	**Source**	**Sensitivity /specificity**	**Technique**	**References**
GTF2A1 and HAAO	Tumor tissue	Presence of malignancy 95/88%; 89/82%	qMSP	Huang et al., 2009
HOXA9 and SCGB3A1	Tumor tissue	Early stage carcinoma (18/24, *p* = 0.002) (5/25, *p* = 0.020)	MSP	Wu et al., 2007
RASSF1A and HIC1	Tumor tissue	Early stage ovarian carcinomas 34/100, 2/68, OR = 0.3 34/100, 10/68, OR = 0.4	MSP	Feng Q. et al., 2008
RASSF1A, GSTP1, MGMT, APC	Tumor tissue	Presence of invasive tumors RASSF1A: 30% vs. 0%; GSTP1: 9% vs. 0%; MGMT: 9% vs. 0%; APC:22% vs. 0%;	MSP	Barton et al., 2008
SPARC	Tumor tissue	Association with tumor gradeMethylation frequency: 68%	MSP	Socha et al., 2009
CDH13, CRABP1, HOXA9, and SCGB3A1	Tumor tissue	Histological subtype differentiation CDH13, CRABP1, HOXA9 and SCGB3A1 (P = 0.041, P <0.001, P = 0.007, P <0.001)	MSP	Wu et al., 2007
CTGF	Tumor tissue	Inversely correlated with invasive disease	cDNA microarray analysis	Barbolina et al., 2009
CCBE1	Tumor tissue	Inversely correlated with metastasis 6/11 (55%) in OC cell lines 38/81 (41%) OC tumors.	Small-interfering RNA (siRNA)-mediated knockdown	Barton et al., 2010
HIC1	Tumor tissue	Presence of malignancy Methylation frequency: 35%	MSP	Rathi et al., 2002
CDH1	Tumor tissue	Inversely correlated with metastasis	Small-interfering RNA (siRNA)-mediated knockdown	Sawada et al., 2008
SFN, TMS1, and WTI	Tumor tissue	Methylation exclusive for Clear cell subtype	MSP	Barton et al., 2008
hMLH1, CDKN2A, and MGMT	Matched tumors	Associated with development of Synchronous endometrial and ovarian cancer Methylation frequency: 39, 41, and 48%	MSP	Furlan, 2006
*14–3-3*s	Tumor tissue	Advanced stage ovarian carcinomas	MSP, quantitative reverse transcription-PCR	Akahira et al., 2004b
HOXA11	Tumor tissue	Presence of malignancy, Suboptimal tumor debulking and poor outcome (relative risk for death = 3.4)	MethyLight assay	Fiegl et al., 2008
10 gene panel	Tumor tissue	Presence of serous adenocarcinoma (69.4% sensitivity and 70.2% specificity)	Microarray Mediated Methylation Assay (MethDet test)	Melnikov et al., 2009
Polycomb group target genes in particular HOXA9	Normal endometrium	Hoxa9 hypermethylation association with increased risk (12.3 fold) of ovarian cancer	MethyLight assay	Widschwendter et al., 2009
SNCG, MASPIN, and CLDN4	Tumor tissue	Advanced stage ovarian carcinomas	Small-interfering RNA (siRNA)-mediated knockdown, qRT-PCR	Gupta et al., 2003; Rose et al., 2006; Choi et al., 2007; Honda et al., 2007
PCDH17	Tumor tissue	Presence of malignancy	Next-generation sequencing, Methylation-sensitive high-resolution melting Analysis	Baranova et al., 2018
EGFL7 and RASSF1	Tumor tissue	Early stage disease detection and progression	DNA methylation microarray assay, Bisulfite pyrosequencing	Rattanapan et al., 2018
LDOC1	Ovarian cancer cell line	Early stage disease detection	RT-PCR and real-time PCR	Buchholtz et al., 2014
GPR150, ITGA8, and HOXD11	Ovarian cancer cell line	Tumor marker	Methylation-sensitive-representational difference analysis (MS-RDA) and MSP	Cai et al., 2007
TGFBI	Tumor tissue and ovarian cancer cell line	Presence of malignancy	Real-time RT-PCR, MS-PCR, and bisulfite sequencing	Kang et al., 2010
DAPK1 and SOX1	Tumor tissue	Early stage disease	MethyLight	Kaur et al., 2016
BORIS	Tumor tissue	Presence of malignancy	qRT-PCR	Woloszynska-Read et al., 2007
long-intergenic non-coding RNA (lincRNA) gene (LOC134466)	Tumor tissue and ovarian cancer cell line	Potential novel diagnostic biomarker for high grade (Type II) serous ovarian carcinoma (HGSOC)	MeDIP-Chip and Sequenom massARRAY methylation analysis	Gloss et al., 2012

Several studies have identified the association of tumor-specific gene methylation with molecular, clinical, and pathological characteristics of epithelial ovarian carcinomas. For instance, highest degree of promoter methylation of SFN (an inhibitor of cell cycle progression), TMS1 and WT1 has been demonstrated in clear-cell ovarian tumors than in other histological types (Kaneuchi et al., 2004, p. 14; Terasawa et al., 2004; Kaneuchi et al., 2005; Teodoridis et al., 2005). Another finding suggests that promoter methylation of RASSF1A, APC, GSTP1, and MGMT correlates with the presence of invasive ovarian carcinomas (Makarla et al., 2005). Hypermethylation of FOXD3 correlated with tumor suppressive role (inhibition of proliferation, migration and promotion of apoptosis) in ovarian cancer cells and thus could serve as a potential therapeutic target for diagnosis of ovarian cancer (Luo et al., 2019).

Using a high–throughput approach to screen genes that showed highest differential methylation between ovarian cancer and normal tissue, Melnikov et al. identified 10 genes to be informative in tissue samples which include: BRCA1, EP300, NR3C1, MLH1, DNAJC15, CDKN1C, TP73, PGR, THBS1, and TMS1. A maximum sensitivity of 69% with 70% specificity was attained on testing the potential of several combinations of these genes to discriminate normal from cancer tissue. Since, all tumors analyzed were of advanced stage (either stage IIIA or higher), therefore, the potential of this panel to diagnose EOC at an early stage is unknown (Melnikov et al., 2009). Ibanez de Caceres et al. demonstrated that hypermethylation of atleast one of the six genes in panel (BRCA1, RASSF1A, APC, p14^arf^, p16^ink4a^, and DAPK) could be detected in 70/ 71 (99%) of EOCs using methylation specific PCR. Furthermore, none of the normal non-neoplastic tissue showed methylation, revealing a specificity of 100%. Additionally, across all histological subtypes, grades, stages as well as age, hypermethylation of TSGs was observed (Ibanez de Caceres et al., 2004). Taken together, these results support hypermethylation of these tumor suppressor genes as a relatively early event in ovarian carcinogenesis and could serve as a potential biomarker for detection and accurate discrimination of EOC at early stage.

Using 7- genes panel [secreted frizzled receptor proteins 1, 2 4, 5 (SFRP1, 2, 4, 5), SRY box1 (SOX1), paired box gene 1(PAX1), and LIM homeobox transcription factor 1, alpha (LMX1A)], Sui et al. investigated methylation in 126 primary ovarian tumors, 75 benign ovarian tumors and 14 borderline ovarian tumors and in 26 OC serum samples. Their findings indicated that promoter methylation of any one of SOX1, PAX1, and SFRP1 could distinguish EOC patients from normal control with a sensitivity of 73.08% and a specificity of 75%. Though these test scores are higher than those of CA125 alone, however it is probably not high enough to warrant its implementation as a diagnostic test for individual patients. Moreover, as no specification of tumor stage within the studied group was provided, the performance of this panel in detection of EOC at an early stage therefore remains unclear (Su et al., 2009).

Hypomethylation induced abnormal expression of several oncogenes such as CLDN4 (encodes an integral component of tight junctions) (Honda et al., 2006; Litkouhi et al., 2007), MAL (mal, T-cell differentiation protein) (Lee et al., 2010), BORIS (a cancer testis antigen family candidate oncogenes) (Woloszynska-Read et al., 2007), and IGF2 (an imprinted gene involved in other malignancies) (Murphy et al., 2006) has been demonstrated in ovarian carcinomas. Promoter hypomethylation induced upregulation of other cancer-associated genes in ovarian cancer includes maspin (SERPINB5) (Rose et al., 2006), MCJ (Strathdee et al., 2004, 2005), and SNCG (synucelin-γ) (Gupta et al., 2003; Czekierdowski et al., 2006b), which encodes an activator of the MAPK and Elk-1 signaling cascades. Hypomethylation of SNCG, MASPIN, and CLDN4 correlates with advanced-stage and metastasis while that of BORIS is linked with disease presence.

Hypomethylation of Sat2 (satellite 2) DNA in the juxtacentromeric region of chromosome 1 and 16 has been reported in ovarian cancer (Qu et al., 1999). A significant increase in hypomethylation of chromosome 1 Sat2 and chromosome 1 satellite α from non-neoplastic tissue toward ovarian cancer tissue was observed. Higher hypomethylation levels were observed in serous and endometrioid tumors in comparison to mucinous. Moreover, extensive hypomethylation was prevalent in high grade or advanced stage tumors (Widschwendter M. et al., 2004). Taken together, consistent higher expression levels along with hypomethylation of L1 and human endogenous retrovirus- W retrotransposons (repetitive sequences widely distributed throughout the genome) has been reported in malignant ovarian tumors against normal control samples (Menendez et al., 2004). It has been hypothesized that promotion in homologous recombination as a result of increased hypomethylation, leads to chromosomal aberrations associated with carcinogenesis (Kolomietz et al., 2002; Symer et al., 2002).

##### Prognosis

Potential prognostic biomarker includes FBXO32, which correlates with advanced stage and shorter disease free survival (Chou et al., 2010), Ribosomal DNA (18S and 28S) linked with prolonged disease free survival (Chan, 2005), IGFBP-3, correlates with disease progression and death in early stage EOC (Wiley et al., 2006b) and HOXA11, association with postsurgical residual tumor and poor outcome (Fiegl et al., 2008). Methylation of ≥1 gene of SFRP1, SFRP2, and SOX1 correlated with short disease free survival while SOX1, LMX1A, and SFRP1 methylation was associated with recurrence and short overall survival (Su et al., 2009). A progression-free survival prediction accuracy of 95% is reported by Wei et al. with hMLH1, IGFP3, and NEUROD1 among a panel of 112 highly discriminatory loci (Wei, 2006). Furthermore, detection of prognostic epigenetic biomarker has also been described in plasma as well as peritoneal fluid. Methylation of hMLH1, analyzed in 138 plasma samples predicted poor survival (hazard ratio: 1.99) (Gifford, 2004) while CDH1, CDH13, and APC (out of a 15 gene panel) analyzed in peritoneal fluid from 57 ovarian cancer patients could predict overall survival (Suehiro et al., 2008). Huang et al. recently reported that the epigenetic loss of heparin sulfate 3-O-sulfation makes ovarian cancer cells sensitive to oncogenic signals and could predict prognosis, thereby reflecting the utility of HS3ST2 for targeted therapy (Huang et al., 2018).

Recently using genome-wide methylation data analysis, five-methylation signature (SLC39A14, PREX2, KCNIP2, CORO6, and EFNB1) were reported as novel independent prognostic biomarker for patients with ovarian serous cystadenocarcinoma, which significantly associated with OS of patients. Moreover, these signatures exhibited higher sensitivity and specificity to predict OSC prognosis (AUC = 0.715), which reflects their clinical significance in improving outcome prediction. Furthermore, these 5- methylation signatures were more accurate over known biomarkers in predicting prognostic survival of OSC patients (Guo T. Y. et al., 2018). Promoter methylation of BRCA1 has been reported to be associated significantly with increased PFS of patients with OC undergoing adjuvant platinum–taxane-based chemotherapy (*P* = 0.008) as well for the patients with disease recurrence (PFS = 18.5 months over 12.8 months for patients without BRCA1 promoter methylation), thereby reflecting that promoter methylation of BRCA1 could be a better predictive marker of response to platinum–taxane-based chemotherapy in sporadic Epithelial ovarian carcinoma (Ignatov et al., 2014).

Another study highlights the potential of CDH1, DLEC1, and SFRP5 gene methylation panel as a prognostic biomarker in advanced stage OC patients. Presence of two or more methylated genes in patients significantly correlated with disease recurrence (hazard ratio: 1.91; *p* = 0.002) and shorter overall survival and disease free survival (hazard ratio: 1.96; *p* = 0.006) (Lin et al., 2018). Liu et al. reported the prognostic potential of C/EBPβ (a transcription factor) which augments chemoresistance of ovarian cancer cells by maintaining an open chromatin state via reprogramming H3K79 methylation of multiple drug-resistance genes upon direct interaction with DOT1L (DNA methyltransferase), thus provides a new insight for more precise therapeutics options in OC by identifying and targeting the key regulators of epigenetics (Liu et al., 2018).

Several recent researches have suggested the hypermethylation and reduced expression is prognostic for shorter progression free survival. For instance, using genome wide array analyses, Hafner et al. reported 220 differentially methylated region with short and long PFS. Validation experiments on a large cohort of type II EOC revealed the association of RUNX3/CAMK2N1 with poor clinical outcome (Lower PFS), indicating the prognostic potential of these genes (Häfner et al., 2016). Few studies have highlighted the tight link between promoter methylation and metastasis. For instance, stimulation of ovarian cancer cell lines by TGFβ, which is a key player in metastasis, extensively change promoter methylation of genes that are associated with EMT (Epithelial-mesenchymal transition) and progression of cancer (Cardenas et al., 2014). Deng et al. reported the tumor suppressive role of IQGAP2 which suppresses the ovarian cancer progression via suppressing Epithelial-mesenchymal transition by regulating Wnt/β signaling, thereby providing a potential biomarker and therapeutic strategy to combat ovarian cancer diagnosis (Deng et al., 2016).

Brachova et al. studied the association of oncomorphic TP53 mutation on patient outcome diagnosed with advanced EOC. Oncomorphic TP53 mutation correlated with worse progression free survival, higher risk of recurrence and higher rate of platinum resistance (Brachova et al., 2015). Dai et al. explored the association of methylation-based prognostic biomarkers within key ovarian cancer-related pathways with progression free survival to platinum based chemotherapy in HGSOC. NKD1, VEGFB, and PRDX2 were identified as the best predictors of progression free survival (PFS: HR = 2.3 *p* = 3.3 × 10–5; Overall Survival: HR = 1.9, *p* = 0.007). Further validation using independent TCGA data set revealed the significant association of VEGFA, VEGFB, and VEGFC promoter methylation with progression free survival (Dai et al., 2013).

Promoter hypomethylation and expression of PRAME correlates with increased survival in high grade serous ovarian carcinoma (Zhang et al., 2016). Promoter hypomethylation and increased expression of proto-oncogenes is predictive for more aggressiveness and metastasis of disease and thereby lower survival, which is evident from recent studies on GABRP, SLC6A12, MGAT3, CT45, CA9, MUC13, and AGR2 (Sung et al., 2014a,b,c, 2017a,b; Zhang et al., 2015; Kohler et al., 2016). Hypomethylation of Sat2 DNA (Chr 1) was associated relapse and poor prognosis (Widschwendter M. et al., 2004), and LINE1 was linked with poorer overall survival and lower progression free survival (Pattamadilok et al., 2008; Table 7).

**Table 7:** Epigenetic biomarkers for ovarian cancer prognostication.

**Epigenetic marker**	**Source**	**Clinical prediction**	**References**
182-gene panel	Tumor tissue	HR 2.5 for PFS	Wei et al., 2002
112-gene panel	Tumor tissue	Prediction accuracy of 95% for shorter Disease free survival	Wei, 2006
*SFRP1, SFRP2*, and *SOX1*	Tumor tissue	Correlates with shorter Disease free survival	Su et al., 2009
*SOX1, LMX1A*, and *SFRP1*	Tumor tissue	Correlates with shorter overall survival	Su et al., 2009
hMLH1	Plasma	If hypermethylated HR:1.99 for OS	Gifford, 2004
HOXA10, HOXA11	Tumor tissue	RR for death:3.4, if HOXA11 methylated	Fiegl et al., 2008, p. 11
18S and 28S rDNA	Tumor tissue	Prolonged DFS	Chan, 2005
EN2	Tumor tissue	Short progression free survival	McGrath et al., 2018
MYLK3	Tumor tissue	Higher methylation level significantly predicted better overall survival with least residual disease	Phelps et al., 2017
FBXO32	Tumor tissue	Advanced stage and short DFS	Chou et al., 2010
Panel of IGFFBP3, p16, BRCA1, GSTP1, ER-α, hMLH1	Tumor tissue	Seven fold increased risk of short DFS HR: 6.53 for disease progression	Wiley et al., 2006a
RUNX3, CAMK2N1	Tumor tissue and ovarian cancer cell line	Short overall survival	Häfner et al., 2016
ABCA1	Tumor tissue and ovarian cancer cell line	Shorter overall survival	Chou et al., 2015
GULP1	ovarian cancer cell line	Residual disease, worse overall survival, and disease specific survival	Maldonado et al., 2018, p. 1
FZD4, DVL1, and ROCK1	Tumor tissue	Correlated with early disease relapse	Dai et al., 2011
**DNA hypomethylated genes**			
15 gene panel	Peritoneal fluid	Short overall survival	Muller, 2004
Sat 2 DNA (Chr1)	Tumor tissue	RR for relapse:4.1, RR for death:9.4 if region methylated	Widschwendter M. et al., 2004
LINE1	Tumor tissue	Lower methylation level significantly predicted poor OS and PFS	Pattamadilok et al., 2008
ATG4A, HIST1H2BN	Tumor tissue	Poor progression free survival	Liao et al., 2014

Another important study by Wei et al. reported 112 methylated loci which were prognostic for reduced PFS and could predict PFS with an accuracy of 95% using Significance Analysis of Microarray and Prediction Analysis of Microarray algorithm (Wei, 2006). Twenty-two hypermethylated loci were identified by global methylation profiling of 485 tumor samples of clear-cell ovarian cancer in a recent study. These hypermethylated loci were associated with 9 genes (VWA1, FOXP1, FGFRL1, LINC00340, KCNH2, ANK1, ATXN2, NDRG21, and SLC16A11). Further, methylation induced silencing of KCNH2 (HERG, a potassium channel) could be a better prognostic factor for poor survival provided increased proliferation was mediated by overexpression of Eag family members. However, further validation on larger cohort is still warranted (Cicek et al., 2013). Huang et al. identified 63 differentially methylated regions of prognostic relevance which significantly correlated with poor PFS. Further, epigenetic silencing of regulators of hedgehog signaling pathway ZIC1 and ZIC4 was associated with increased proliferation, migration, and invasion. Additionally, promoter hypermethylation of ZIC1 significantly correlated with poor survival and thus could serve as prognostic determinant for patient outcome (Huang et al., 2013).

Another study describes that the global methylome status of HGSOC PDX (patient-derived xenografts) resembled with global methylation in corresponding patient tumor over several generations and could be efficiently modulated by demethylating agents. C-terminal Src kinase (CSK), a novel epigenetically regulated gene and associated pathways were also identified. Low CSK methylation significantly correlated with improved PFS and OS in HGSOC patients (Tomar et al., 2016). Koestler et al. using integrative global methylation and single nucleotide polymorphisms analysis identified DNA methylation marks (13 unique CpGs and 17 unique SNPs) which could mediate EOC genetic risk (Koestler et al., 2014).

Recently, Sharma et al. investigated epigenetic regulation of POTE gene family, which is localized to autosomal pericentromeric region. POTE gene family is over-expressed in HGSOC. Epigenetic silencing of POTE gene was functionally verified by experiments involving treatment with Decitabine and DNMT knockout cell lines. In addition expression of individual gene in POTE gene family correlated with chemoresistance and poor clinical outcome in HGSOC patients. Furthermore, several epigenetic alternations (pericentromeric activation, global and locus-specific L1 hypomethylation, and locus-specific 5' CpG hypomethylation) served as a determinant for regulation of epigenetic activation of POTE gene (Sharma et al., 2019).

In conclusion, these studies provides insight to the association of several potential methylation based prognostic biomarkers with clinical outcome in ovarian carcinoma and further suggest that these reports on epigenome wide interrogation of DNA methylation warrants detailed functional analysis of loci sufficiently discriminating OC with normal state. New targets identified through comprehensive methylome analysis in OC have significant translational potential to pave the design of future clinical investigations and therapeutics.

##### Predictive

Methylation mediated transcriptional repression of specific drug-response genes results in acquisition of drug resistance and significantly extends its impact on different facets of chemotherapeutic actions: membrane entry/exit, drug metabolism, response to cellular injury, DNA repair, apoptosis etc., in cancer cells. Hypermethylated genes such as hMLH1, ASS1 (arginine biosynthesis-related gene), ESR2 (encoding ER-β), and SFRP5 (encodes an inhibitor of oncogenic WNT signaling pathway) have been implicated in platinum resistance. Three studies well defined in ovarian cancer includes: Methylation of either BRCA1, GSTP1, or MGMT significantly correlates with improved response to chemotherapy (*p* = 0.013) (Teodoridis et al., 2005). Hypermethylation of RASSF1A and CABIN1 have been reported to correlate with response to adjuvant therapy. Patients who responded to therapy had moderately higher frequencies of RASSF1A hypermethylation (OR = 0.4) and significantly higher frequencies of CABIN1 hypermethylation (OR = 0.1) (Feng Q. et al., 2008). Strathdee et al. demonstrated that high levels of MCJ methylation significantly correlated with poor response to therapy (p = 0.027) and poor overall survival (*p* = 0.023; HR = 2.9) (Strathdee et al., 2005). Hypomethylation induced upregulation of ABCG2 (multidrug transporter) MAL (determinant of platinum resistance) and TUBB3 (determinant of taxane resistance) genes have been described in advanced ovarian carcinoma cases with drug-acquired chemoresistance (Izutsu et al., 2008; Balch et al., 2010; Lee et al., 2010; Table 8).

**Table 8:** Epigenetic biomarkers for ovarian cancer prediction.

**Epigenetic marker**	**Source**	**Clinical prediction**	**References**
Methylation of >1 of BRCA1, GSTP1, and MGMT	Tumor tissue	Association with improved response to chemotherapy	Teodoridis et al., 2005
RASSF1A, CABIN1	Tumor tissue	Association with response to chemotherapy	Feng Q. et al., 2008
ASS1	Tumor tissue	Determinant of Platinum resistance	Nicholson et al., 2009
HSulf1	Tumor tissue	Determinant of Platinum resistance	Staub et al., 2007
SFRP5	Tumor tissue	Determinant of Platinum resistance	Su et al., 2010
hMLH1	Plasma	Relapse of Chemoresistant tumor	Gifford, 2004
ESR2	Tumor tissue	Determinant of Platinum resistance	Yap et al., 2009
MCJ	Tumor tissue	Association with response to chemotherapy and overall survival	Strathdee et al., 2005
TUBB3	Tumor tissue	Taxane resistance	Izutsu et al., 2008
MSX1	Ovarian cancer cell line	Sensitivity to platinum drug	Bonito et al., 2016
TBX2	Tumor tissue	Sensitivity to platinum drug	Tasaka et al., 2017
MAL	Tumor tissue	Platinum resistance	Lee et al., 2010

Recently Pulliam et al. demonstrated the combinatorial effect of DNA methyltransferase inhibitor (DNMTi) guadecitabine and the Poly (ADP-ribose) polymerase (PARP) inhibitors (PARPi) talazoparib in resensitizing PARPi resistant breast and ovarian cancer irrespective of BRCA status. Synergistic effect of guadecitabine and talazoparib increased ROS accumulation, and further sensitized the breast and ovarian cancer cells toward PARPi sensitivity by subsequent activation of cAMP/PKA signaling which in turn promoted PARP activation. Furthermore, DNMTi augmented PARP “trapping” by talazoparib. The finding of this complementary model supports further clinical exploration of this combination therapy in PARPi-resistant cancers (Pulliam et al., 2018). Another study using integrated global methylation analysis on extreme chemoresponsive HGSOC patients identified four genes of clinical relevance (FZD10, FAM83A, MYO18B, and MKX) as epigenetic marker of platinum based chemoresponse, of which, FZD10 was reported as functionally validated marker of platinum sensitivity (Tomar et al., 2017). Promoter methylation of OPCML was significantly associated with poor overall survival of OC patients and thus could be of use in predicting disease prognosis (Zhou et al., 2014).

A recent study has described induction of hypomethylation in resistant ovarian cancer patients upon treatment with cisplatin, though, in the intergenic regions, the loss of methylation was primarily observed (Lund et al., 2017). Hypomethylation of developmental genes MSX1 and TMEM88 correlated with platinum resistance in patients with ovarian cancer (Bonito et al., 2016; de Leon et al., 2016). Stimulation of EMT by non-coding RNA HOTAIR has been reported to be regulated by DNA methylation and is indicative of resistance to carboplatin (Teschendorff et al., 2015). Likewise, another study highlights promotion of platinum resistance by TET. Induction of EMT by TET is mediated by demethylation of Vimentin promoter in ovarian carcinoma (Han et al., 2017).

A recent study has described how methylome-targeting strategies could bring forth anti-tumor effect. Guadecitabine-mediated induction of global hypomethylation not only affects metabolic and immune responses but also activates tumor suppressor genes which eventually contribute to platinum drug re-sensitization in ovarian cancer. This might offer utility in improving survival outcomes of patients with ovarian cancer (Fang et al., 2018). Another recent study has highlighted the tumor suppressor role of ZNF671 and its methylation could act as a predictor for early recurrence of serous ovarian carcinoma (Mase et al., 2019). Another important study by M. Keita et al. has for the first time reported the exclusive association of massive DNA hypomethylation with poorly differentiated tumors, which correlates with disease aggressiveness and progression. This report also raises concern over the adverse effect of use of demethylating agents which probably aid the activation of oncogenes and prometastatic genes (Keita et al., 2013).

In conclusion, it is speculated that the combinatorial therapies utilizing epigenetic inhibitors holds promise and would be most effective for chemo-resensitization of resistant tumors, possibly by restoration of pathways associated with drug response, and thus would subsequently implicate improved survival outcomes as well as personalized treatment for this devastating disease.

#### Histone Modifications in Ovarian Cancer

Compared with DNA methylation, the evidence on chromatin modification in development of ovarian cancer is limited. Histone modification mediated regulation of cell cycle regulatory proteins such as cyclin B1 (Valls et al., 2005), p21 (Richon et al., 2000), and ADAM19 (Chan M. W. et al., 2008) have been described in various reports. Association of histone modifications with aberrant class III β tubulin protein expression (Izutsu et al., 2008), reduction of PACE3 expression (Fu et al., 2003) and silencing of survivin (Mirza et al., 2002) has been reported in ovarian tumorigenesis. Upregulation of tumor suppressor Rb and CDKN1 (cyclin-dependent kinase inhibitor) by histone acetylation was described by Strait et al. (2002). Moreover, the overexpression of HDACs 1–3 in ovarian cancer has been reported to be associated with high grade tumors and resulting poor prognosis (Weichert et al., 2008). On the other note, the derepression of claudin-3 and claudin-4 was found to be associated with loss of trimethylated histone 3 lysine 27 (H3K27me3) (Kwon et al., 2010). The transcriptional repression of osteoprotegerin (OPG) has been reported to be mediated by reduced histone 3 lysine 4 trimethylation (H3K4me3) and increased H3K27me3 (Lu et al., 2009). Similarly, the association of transcriptional silencing of GATA4 and GATA6 with hypoacetylation of histones H3 and H4 and loss of trimethylated histone 3l ysine 4 (H3K4me3) has been described by Caslini et al. (2006).

A very recent report has provided insight into the mechanism associated with development and progression of OC. Early Loss of E3 ubiquitin ligase RNF20 and histone H2B monoubiquitylation (H2Bub1) has been reported to drive ovarian tumorigenesis by altering chromatin accessibility and thereby activating immune signaling pathways (IL6), and this loss has been defined by majority of high grade serous ovarian carcinomas tumors (Hooda et al., 2019). Cacan et al. reported that the loss of FAS expression which contributes to drug resistance is mediated by histone deacetylase 1 (HDAC1) in chemoresistant OC cells (Cacan, 2016). Recently Tang et al. highlighted the repression of histone H3 lysine 27 trimethylation (H3K27me3) which was mitigated by AMP-activated protein kinase (AMPK) phosphorylation upon treatment with metformin thus implicated the antitumor effect of metformin and suggested its utility in the treatment of EOC patients who are not diabetic (Tang et al., 2018).

In another study, the mechanism associated with upregulation of ABCB1 was conferred to chromatin remodeling (via p300 mediated H3K9ac and AR complex binding to ARE4) which in turn leads to the development of taxol resistant phenotype. It was shown that the upregulation of p300 and GCN5 (HATs) was associated with overexpression of ABCB1 and resistance to taxol and PI3K/AKT pathway which is activated by taxol, mediates the regulation of the expression of p300 and AR. These results further reveal the significance of AKT/p300/AR axis as a novel treatment strategy in combating taxol resistance (Sun et al., 2019). Using ChIP-seq approach, Curry et al. identified genome-wide bivalent domains (H3K27me3 and H3K4me3) at gene promoter in tumor samples which were collected pre and post platinum resistance acquisition, and showed that these representative poised gene sets are pre-disposed to hypermethylation induced epigenetic silencing during acquisition of drug resistance, thus provides novel insights to prevent emergence of drug resistance (Curry et al., 2018).

Yi et al. reported that Enhancer of zeste homolog 2 (EZH2) mediates repression of tissue inhibitor of metalloproteinases 2(TIMP2) by H3K27me3 and DNA methylation thereby facilitating ovarian cancer metastasis (Yi et al., 2017). In similar context, another study highlighted silencing of ARHI in ovarian cancer which was synergistically mediated by Enhancer of zeste homolog 2 (EZH2) induced H3K27me3 and DNA methylation. Furthermore increased EZH2 expression correlated with worse overall survival rates, implicating prognostic potential of EZH2 in EOC (Fu et al., 2015). Repression of Regulator of G-protein signaling 2 (RGS2) via histone deacetylases (HDACs) and DNA methyltransferase I in chemoresistant OC cells has been reported recently by Cacan et al., and utility of their inhibitors might serve as a novel approach to overcome chemoresistance in ovarian cancer (Cacan, 2017).

### Clinical Application of Epigenetic Biomarker in Liquid Biopsies for Ovarian Cancer Management

#### Cell Free DNA Biology

Advancement in the understanding underlying molecular pathogenesis of cancer, along with advancements in molecular techniques has facilitated the study of molecular alternations associated with cancer development at an early stage in body fluids. Circulating cell free DNA which are believed to have derived from tumor cells, reflect specific genetic and epigenetic alternations, and thus may offer potential non-invasive viable biomarkers for several cancer, capable of providing valuable information regarding disease progression and response to therapy in real time.

**Figure 3:**
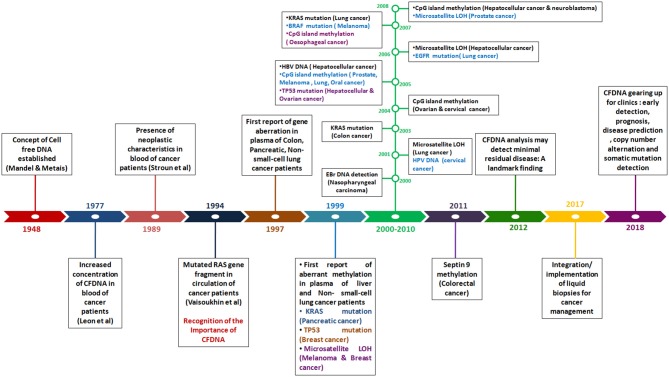
Timeline reflecting the detection of genetic and epigenetic alternations in Cell-free DNA in blood of patients with different cancer type.

In 1948, the existence of cell free DNA was first described by Mandel and Métais. Cell free DNA are derived from necrotic and apoptotic cells, commonly released by all cell types. Further, numerous subsequent studies confirmed that the tumor-specific pattern of alterations, such as chromosomal abnormality, somatic mutations, resistance mutation, aberrant methylation and copy number variations could be found in cfDNA, which can serve as potential target for diagnosis of cancer through non-invasive approach (Leon et al., 1977; Polivka et al., 2015; Figure 3).

**Figure 4:**
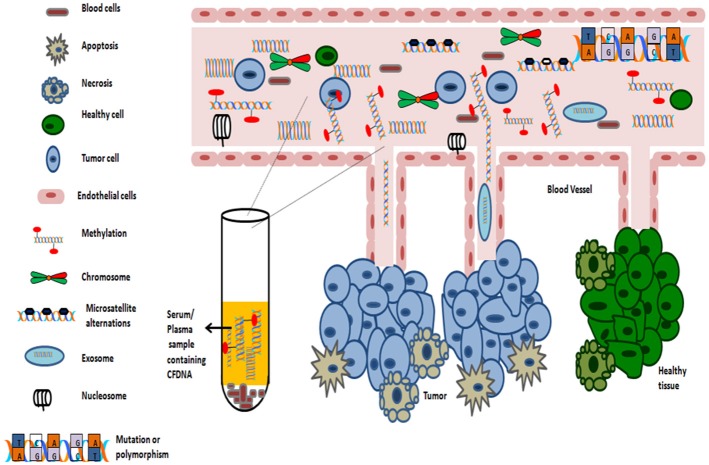
Origin of Cell-free DNA in blood.

Numerous studies support the detection of methylation signature in almost any body fluid (such as serum, plasma, smears, nipple fluid aspirate, and vaginal fluid etc.). As sampling of blood can be considered as minimal invasive process, thus serves as an ideal substrate for methylation analysis. The average concentrations of circulating cell free DNA in healthy subjects is 30 ng/ml. However, in cancer patients, the average concentration of cell free serum DNA is higher, approximately 180 ng/ml as dying cancer cells release tumor DNA into the blood (Gormally et al., 2007). The average length of circulating cfDNA, which are usually fragmented, is 140 to 170 bp and of which, only a fraction of few thousand amplifiable copies of cfDNA /ml of blood, might be of diagnostic relevance (Gormally et al., 2007; Polivka et al., 2015). The levels of circulating cell free DNA in serum is abnormally high in early as well as advanced-stage tumors (Perlin and Moquin, 1972; Leon et al., 1977). For this phenomenon, the proposed two primary mechanisms includes: either cells in cancer tissue undergoes *in situ* apoptosis and/or necrosis or cells might detach from tumors and extravasate into bloodstream where they undergo lysis (Figure 4).

Since its first validation, the potential application of circulating DNA in research settings and for non-invasive management of cancer as “liquid biopsy” is expanding with improvement in molecular and genomic techniques. Numerous studies have demonstrated that tumor specific aberrant methylation can also be detected in cfDNA of patients with different tumor types such as lung, prostate, breast and colorectal cancer and further confirmed altered methylation as an independent diagnostic/ prognostic marker (Board et al., 2008; Brock et al., 2008; Lofton-Day et al., 2008; Vlassov et al., 2010). Warren et al. developed a highly sensitive non-invasive test for screening of colorectal cancer based on methylation of SEPT9 in plasma which could specifically detect all stages and locations of colorectal cancers (Warren et al., 2011). Hypermethylation of Vim gene is strongly correlated with the occurrence of colorectal cancer. Similarly hypermethylation of SHOX2 in sputum has been used as biomarker for distinguishing malignant and benign lung diseases (Kneip et al., 2011). Gstp1 methylation status in urine is strongly correlated with early onset of prostate cancer (Belinsky, 2004).

Numerous reports have highlighted the potential of DNA methylation based biomarkers for non-invasive detection of cancer utilizing cell free DNA. Recently, using integrated methylome analysis Wei et al. reported hypermethylation of SPG20, a putative STAT3 target, for non-invasive detection of gastric cancer at an early stage (Wei et al., 2019). Yang et al. explored the potential of eight gene panel for non-invasive detection of lung cancer using qMSP and revealed that the promoter methylation of any of the eight gene could detect the disease with a sensitivity of 72% with 91% specificity, reflecting the utility of plasma DNA methylation as a novel approach for detection of lung cancer at early stage (Yang et al., 2018).

Similarly, promoter methylation of OPCML and HOXD9 assessed in serum cell free DNA using methylation-sensitive high-resolution melting, was detected with a sensitivity of 62.50% with specificity of 100%, thus could serve as a non-invasive differential biomarker to prevent misdiagnosis of cholangiocarcinoma (CCA) and other biliary diseases (Wasenang et al., 2019). Further, for the management of pancreatic cancer and its early detection Eissa et al. analyzed the methylation of ADAMTS1 and BNC1 in cfDNA using qMSP, which exhibited a sensitivity of 94.8% and specificity of 91.6% with a AUC of 0.95 reflecting diagnostic potential of this blood based two-gene panel in detection of pancreatic cancer at an early stage (Eissa et al., 2019). Methylation of APC, FOXA1, and RASSF1A in cell free DNA served as a best performing cassette in terms of diagnostic and prognostic value, revealing a sensitivity, specificity and accuracy over 70% suggesting its putative utility in management of breast cancer (Salta et al., 2018).

Other studies using genome-wide methylation profiling of serum/plasma cell-free DNA have identified potential biomarkers for clinical utility. For instance, Xu et al. using MeDIP-seq approach reported 10 significant differentially methylated genes as potent biomarker for lung cancer clinical application (Xu et al., 2019). Similarly, using genome-wide methylome profiling and Sequenom MassARRAY approach, it was reported that promoter methylation of CASZ1, CDH13, and ING2 could serve as a potent noninvasive biomarker for detection of esophageal cancer at early stage (Wang H.Q. et al., 2018).

#### Challenges

The analysis of blood borne cell-free DNA has tremendous potential to enable rapid, non-invasive molecular diagnosis of cancer. They are of great clinical relevance as they provide specific targets for initial diagnosis, permit monitoring of treatment efficacy as well as information about tumor profile and its dynamics which are critical for treatment decisions (De Mattos-Arruda et al., 2014; Lewis et al., 2015).

The advantages of analyzing tumor specific DNA methylation in cell free serum DNA includes, improved sensitivity as cfDNA can be easily amplified by PCR, fewer false positive rate as methylation pattern is generally conserved throughout the progression of disease, stability during sample collection as abnormal DNA methylation is chemically as well as biologically stable and remains relatively unaffected by physiological condition at the time of sample collection, increased technical sensitivity and specificity for gene specific assays as well as offers assay design advantages over genetic alternation that might be interspersed throughout a given gene. Furthermore, DNA methylation is a positively detectable signal, unlike a loss of signal as in chromosomal deletions (Wittenberger et al., 2014).

Several limitations in the methylation detection of cell free serum DNA includes extremely low amount of available cfDNA, missing bisulfite conversions as they are usually fragmented, low sensitivity demonstrated by a single marker and time-consuming, complicated and expensive conventional techniques for cfDNA isolation. The most commonly used technique for methylation detection is MSP PCR (methylation specific PCR) which is a bisulfite-conversion- based method. The limitation of bisulfite conversion of cfDNA is the missing DNA. Because of the technical difficulties of DNA methylation analysis, only few DNA methylation based markers has been identified to date, which apply only to a fraction of gynecological cancers including breast, ovarian and endometrial cancers (Wittenberger et al., 2014; Lewis et al., 2015).

The two technological challenges to be addressed include (1) the detection of low abundant tumor-specific DNA methylation patterns through methylation specific PCR priming or probing with high signal-to-noise ratio (2) the determination of methylation status of consecutive sites in individual DNA molecules with single base-pair resolution. This requires methylation-independent priming and sequence analysis of combined PCR product. Clinically the major problem associated with DNA methylation assays is to detect scarcely abundant alleles within high background levels of non-target molecules. However, with the advent of digital MethyLight assay together with rapid advances in next generation sequencing based technologies, these issues can be overcome. One example of this novel approach is the development of the PraenaTest™ (LifeCodexx, Germany) (Weisenberger et al., 2008).

#### Serum Based Epigenetic Biomarker

Tumor-specific methylation-based biomarkers might possibly prove valuable for monitoring disease prognosis and different pathological determinants; however, non-invasive analysis and characterization of biomarkers in body fluids offers more feasibility in early screening and detection of the disease as well in monitoring the response to therapy. Numerous studies have reported aberrant methylation in ovarian cancer as discussed earlier; there are relatively few reports of serum/plasma methylation biomarkers for earlier detection of OC. Various studies that demonstrated striking detection sensitivities and specificities in non-invasive assays, thereby supporting the promising utility of these biomarkers for early screening and detection of OC has been summarized in Table 9.

**Table 9:** Non-invasive epigenetic DNA methylation biomarkers for ovarian cancer.

**Epigenetic marker(s)**	**Source**	**Patient/sample**	**Clinical prediction**	**Technology**	**References**
Methylation of ≥1 gene of BRCA1, RASSF1A, APC, p14ARF, p16INK4A, or DAPK	Serum and cytologically negative peritoneal fluid	50 serum, 40 peritoneal fluid from EOC patients along with 40 control serum and peritoneal fluid samples	Presence of malignancy Sensitivity: 41/50 (82% for serum) 28/30 (93% for peritoneal fluid), Specificity: 100% (all tumor stages).	Methylation-specific-PCR (MSP)	Ibanez de Caceres et al., 2004
DAPK	Whole peripheral blood DNA	26 peripheral blood samples	Sensitivity: 14/16 (54%) for DAPK-methylation-positive samples: Specificity: 10/10 (100%) for DAPK methylation negative	Methylation-specific PCR (MSP)	Collins et al., 2006
Methylation of ≥1 gene of SOX1, PAX1 or SFRP1	Serum	46 (26 ovarian cancer cases and 20 patients with a benign condition	Sensitivity: 73.08% Specificity: 75%	Methylation-specific PCR (MSP)	Su et al., 2009
Methylation of 7-gene panel (APC, RASSF1A, CDH1, RUNX3, TFPI2, SFRP5, and OPCML	Serum	202 patients (87 EOC cases, 53 benign cases and 62 controls)	Sensitivity of 7-gene panel: 85.3% Specificity of 7-gene panel: 90.5% in stage I EOC Sensitivity of CA125: 56.1% Specificity of CA125: 64.15%	Multiplex methylation-specific PCR (MSP)	Zhang et al., 2013
10-gene panel (Combination of BRCA1, HIC1,PAX5,PGR, THBS1)	Plasma	66 (33 cancer cases and 33 control)	Presence of malignancy Sensitivity: 61% Specificity: 85%	Microarray based multiplex assay(MethDet56 technique)	Melnikov et al., 2009
Several gene panel (RASSF1A and PGR-PROX) (RASSF1A, CALCA and EP300)	Serum	90 (30 EOC cases, 30 cases with Benign disease along with 30 controls non neoplastic samples)	Methylation of RASSF1A and PGR-PROX Sensitivity: 80.0% Specificity:73.3% Methylation of RASSF1A, CALCA and EP300 Sensitivity: 90.0% Specificity: 86.7%.	Microarray based Assay(MethDet 56)	Liggett et al., 2011
OPCML	Serum	194 (71 EOC, 43 benign and 80 controls non neoplastic samples)	Sensitivity: 87.18% Specificity: 93.75% Accuracy: 90.14%	Nested Methylation -specific PCR (MSP)	Wang et al., 2017
RASSF1A	Plasma	53 samples including OC tumors, adjacent tumor cell free tissues and paired plasma circulating tumor DNA	Sensitivity: 33/53 (62.3%), RASSF1A methylation of paired plasma CtDNA showed slight concordant with primary tumor samples (*P* = 0.227, 2-sided Pearson χ2 test, *k* = 0.156). Significantly correlates with overall survival	Real-time methylation specific-PCR (real-time MSP) and methylation-sensitive high-resolution melting analysis (MS-HRMA)	Giannopoulou et al., 2017
ESR1	Plasma	Group A: 66 OC cases Group B: 53 OC case along with 50 paired plasma samples	Sensitivity of detection: 38%. ESR1 methylation predicted better clinical outcomes: overall survival (*P* = 0.027), progression-free survival (*P* = 0.041)	Real-time methylation-specific PCR (real-time MSP) assay	Giannopoulou et al., 2018
3-gene panel	Serum	For assay development: 151 cases and for validation study 250 cases with different conditions in 3 sets	Pre-chemotherapy Sensitivity: 41.4% Specificity: 90.7% Post chemotherapy Responders: 78% non-responders: 86%	Targeted ultra-high coverage bisulfite sequencing	Widschwendter et al., 2017

### MicroRNAs in Ovarian Cancer

Aberrant expression of microRNAs has been confirmed in ovarian carcinogenesis. A decrease in mRNA levels of the miR- processing enzymes in OC malignant cases against normal controls, strongly implicates an overall tumor suppressive role of miRs in ovarian tumorigenesis (Merritt et al., 2008; Pampalakis et al., 2010). Overexpression of Drosha and Dicer was significantly associated with better survival, while low expression of Drosha was associated with suboptimal surgical cytoreduction and low expression of Dicer with advanced tumor stage, thereby further implicating the tumor suppressive role of microRNAs in OC (Merritt et al., 2008; Faggad et al., 2010). With respect to ovarian cancer, the potential targets for several upregulated miRs includes pro-apoptotic, metastasis-suppressing or antiproliferation gene products while those for the downregulated miRs includes growth signaling, prometastatic- or anti-apoptosis-associated proteins. A list of upregulated/downregulated miRs involved in ovarian cancer development is shown in Table 10. A list of aberrantly expressed miRs which could serve as a promising biomarker for detection of ovarian cancer has been summarized in Table 11. Chao et al. reported that in advanced stage cancer, miR-187 regulates carcinogenesis through Dab2 dependent EMT (epithelial-to-mesenchymal transition) (Chao et al., 2012, p. 2). Furthermore, other studies have described miR-199a, miR-200a, miR-200b, miR-200c, and miR-214 as significantly overexpressed and miR-100 and miRNAlet-7i as significantly downregulated in ovarian tumors (Iorio et al., 2007; Yang H. et al., 2008; Yang N. et al., 2008). Several miRNA signatures that could distinguish ovarian tumors based on histological subtypes has been studied such as miR-200b and miR-141 was observed to be overexpressed in serous and endometrioid subtypes; upregulated of miR-21, miR-203, and miR-205 correlated with endometrioid histotype; downregulated miR-145 correlated with serous and clear cell subtype, while downregulated miR 222 was associated with endometrioid and clear cell subtype (Iorio et al., 2007).

**Table 10:** List of dysregulated miRNAs in ovarian cancer.

	**Mechanism(s)**	**miR(s)**	**Targets**	**Consequence(s) leading to tumorigenesis**	**References**
Upregulated	c-myc activation	130a	MCSF, GAX and HOXA5	Chemotherapy resistance, angiogenesis, and dedifferentiation	Taylor and Gercel-Taylor, 2008; Eitan et al., 2009
	Copy gain	27a and 451	ZBTB10, Myt-1, HMGB2, HOXA2, and CYP1B1	Multidrug export, oncogenic signaling and reduced apoptotic potential	Shibata et al., 2006; Zhang L. et al., 2006
		213	APP and SATB2	Chemoresistance	Boren et al., 2009
		199a, 200a, b, c and 335	TGFβ, ZEB1, ZEB2, BAP1, GATA4, GATA6, TNC, FN1, EXOC5, and TUBB3	Mesenchymal–epithelial transition	Weisenberger et al., 2008; Su et al., 2009; Liggett et al., 2011; Giannopoulou et al., 2017; Wang et al., 2017
	Hypomethylation	203	p63, SOCS-3, ABL1, MCEF, and ADAMTS6	Unknown	Iorio et al., 2007; Lee et al., 2009
		205	ZEB1, ZEB2, E2F1, ERBB3, PKCe, and SHIP2	Mesenchymal–epithelial transition, oncogenic signal transduction	Iorio et al., 2007; Yu et al., 2008; Lee et al., 2009
		21	PDCD4†, RPS7†, NCAPG†, TPM1, and PTEN	Reduced apoptotic potential and anchorage independence	Iorio et al., 2007; Laios et al., 2008; Sorrentino et al., 2008; Eitan et al., 2009; Lee et al., 2009; Resnick et al., 2009; Yang et al., 2009
	Unknown	340	PAM, RTN3, PPL, RNF34, and ZNF513	Chemoresistance	Boren et al., 2009
		221/222	KIT, AIP1, p21, p57, TCF12, RIMS3, and ARNT	Cell cycle progression and angiogenesis	Merritt et al., 2008; Vlassov et al., 2010; Kneip et al., 2011
Downregulated	C/EBPα Downregulation	1	FOXP1, HDAC4 c-Met, Pim1 and HAND2	Tumor growth, cell motility and proliferation	Iorio et al., 2007
	DNA methylation and copy loss	137	CDK6, MITF, KLF12, and PDLIM3	Cell cycle progression and dedifferentiation	Iorio et al., 2007; Zhang L. et al., 2008
		140	c-SRK, MMP13 and FGF2	Oncogenic signaling	Iorio et al., 2007; Zhang L. et al., 2008
		150	c-Myb, MAK9, Akt3, and MAP2K4	Oncogenic signaling	Zhang L. et al., 2008
		551a	LPHN1, ERBB4, and ZFP36	Oncogenic signaling	Dahiya et al., 2008
		9	NF-kB^†^, Bcl2^†^, Bcl6, FGF^†^, and b-Raf	Oncogenic signaling	Leon et al., 1977; Weisenberger et al., 2008; Faggad et al., 2010; Kneip et al., 2011
		184	TTK69, K10, and Sax(A)	Dedifferentiation	Zhang L. et al., 2008
	Unknown	30b and d	CTGF	Invasion/metastasis	Laios et al., 2008
		98	HMGA2, LIN28B, and HIC2	Oncogenic signaling and cancer stemness	Dahiya et al., 2008
		517a and b	CREAP-1, MAPKAPK5, NFKBIE, and PTK2B	Chemoresistance and oncogenic signaling	Lee et al., 2009
		Let-7i	HMGA2, LIN28Bm TRIM71, and IGF2BP1	Chemoresistance	Yang N. et al., 2008
		662	NEGR1, MKX, and CSF3	Unknown	Dahiya et al., 2008

**Table 11:** List of misexpressed miRNAs in ovarian cancer.

**Epigenetic marker**	**Alternations in miRNAs**	**Source**	**Methodology**	**Clinical prediction**	**References**
Dicer, Drosha mRNA	Downregulated	Tumor tissue and ovarian cancer cell lines	Quantitative RT-PCR, gene expression array	Advanced tumor stage and Suboptimal tumor debulking	Merritt et al., 2008; Faggad et al., 2010; Pampalakis et al., 2010
let-7i, miR-221,−30c,−152 and−193 miR-185,−106a,−181a, −210,−423,−103,−107 and let-7c	Downregulated Upregulated	Tumor tissue from Endometrial cancer, normal endometrial and atypical hyperplasia	Quantitative RT-PCR, microarray analysis	Association with Endometrial cancer development	Boren et al., 2008
miR-124-1,−124-2,−124-3,−127,−132,−137,−193A, 375 and−339	Downregulated	Tumor tissue	Quantitative RT-PCR, MSP, direct Sanger sequencing	EOC metastasis (including peritoneal macro-metastases)	Loginov et al., 2018
let-7i	Downregulated	Tumor tissue and ovarian cancer cell lines	miRNA Microarray, Stem-loop real-time RT-PCR (TaqMan miRNA assay)	Associated with increased resistance to chemotherapy drugs, cis-platinum, and short progression-free survival	Yang N. et al., 2008
miR-30c,−130a, and−335	Downregulated	Paclitaxel and cisplatin resistant cancer cell lines	miRNA Microarray, qPCR, Northern blots	Association with development of chemoresistance	Sorrentino et al., 2008
miR- 199b-5p	Downregulated	Cisplatin-sensitive and -resistant ovarian cancer cell lines	miRCURY LNA^TM^ microRNA array and Q-PCR	Development of acquired chemoresistance through the activation of JAG1-Notch1 signaling cascade	Liu et al., 2014
miR-34a, miR-34b*/c	Downregulated	Tumor tissue	Quantitative RT-PCR, *in- situ* hybridization	Associated with motility and invasion by regulation of MET, progression of disease to advanced stages	Corney et al., 2010
miR−34a,−200a,−200b,−449b,−509-3p,−509-3-5p,−513a-5p and −574-5p	Upregulated	Tumor tissue	MicroRNA microarray	Differentially expressed in Stage I disease	Eitan et al., 2009
miR-302b,−22, and−373 miR-148b and−211	Upregulated Downregulated	Tumor tissue and ovarian cancer cell lines	MicroRNA microarray, Quantitative Real time PCR (Taqman based)	Discriminates serous vs. non-serous disease	Iorio et al., 2007
miR-7, 34c-5p, 146b-5p and 449a	Upregulated	Tumor tissue	Massively parallel pyrosequencing, TaqMan® qRT-PCR assays	Serous adenocarcinoma	Wyman et al., 2009
miR-23p,−125a-3p,−125a-5p,−130a,−146b-5p,−193a-3p,−193a-5p,−423-5p,−451 and−491-5p	Upregulated	Tumor tissue	MicroRNA microarray	Differentially expressed in Stage III disease	Eitan et al., 2009
miRs 100, 199a, 200a, and 214	Upregulated	Tumor tissue and ovarian cancer cell line	miRNA array and Northern blot analysis, quantitative reverse transcription-PCR.	Late clinical stage and high-grade tumors, negative regulation of PTEN by miR-214 thereby inducing cell survival and cisplatin resistance	Yang H. et al., 2008
miR-302b,−325,−299-5p,−222, and−324-3p miR-212 and−150	Upregulated Downregulated	Tumor tissue and ovarian cancer cell lines	MicroRNA microarray, Quantitative Real time PCR (Taqman based)	Differentiates Serous vs. endometrioid disease	Iorio et al., 2007
miR-325,−22,−302c,−299–5p,−373, and−196b miR-9 and−18	Upregulated Downregulated	Tumor tissue and ovarian cancer cell lines	MicroRNA microarray, Quantitative Real time PCR (Taqman based)	Associated with Poor differentiation	Iorio et al., 2007
miR-30a,−30a*, and−486-5p	Upregulated	Tumor tissue and ovarian cancer cell lines	MicroRNA microarray, Quantitative Real time PCR (Taqman based)	Clear cell disease	Iorio et al., 2007
Methylation of let-7a-3	Downregulated	Tumor tissue	Real-time methylation-specific PCR and real-time reverse Transcription-PCR, direct Sanger sequencing	Favorable prognosis	Lu et al., 2007, p. 3
miR-449b	Upregulated	Tumor tissue	MicroRNA microarray	Good prognosis	Eitan et al., 2009
let-7e	Upregulated	Paclitaxel and cisplatin resistant cancer cell lines	miRNA Microarray, qPCR, Northern blots	Associated with resistance to Paclitaxel	Sorrentino et al., 2008
miR-200a,−200b, and−429	Upregulated	Primary tumor and ovarian cancer cell line	Real-time reverse transcription-PCR	Long disease free survival and delayed recurrence, prognostic marker in advanced ovarian cancer	Cochrane et al., 2009; Hu et al., 2009
miRs 100, 199a, 200a, and 214	Upregulated	Tumor tissue and ovarian cancer cell line	miRNA array and Northern blot analysis, quantitative reverse transcription-PCR.	Late clinical stage and high-grade tumors	Yang H. et al., 2008
Methylation of miR-34a	Downregulated	Tumor tissue	Quantitative reverse transcription-PCR, MethyLight assay	Inversely associated with grade, p53 mutation, and dualistic tumor type. Reduced progression free survival and worsen overall survival.	Schmid et al., 2016
Methylation of miR-199a-3p	Downregulated	Tumor tissue and ovarian cancer cell line	Methylation-specific PCR and bisulphite sequencing	Tumor aggressiveness and enhanced cisplatin resistance through promoting DDR1 expression	Deng et al., 2017
Methylation of 10 miRNA genes (miR-124-2,−124-3,−125B-1,−127,−129-2,−137, −193A,−203A,−339,−375)	Downregulated	Tumors tissue and matched peritoneal metastases	Methylation-specific PCR	Involved in metastasis	Loginov et al., 2018
(miR-34b/c, miR-9-1, miR-124-3, miR-129-2, and miR-107)	Downregulated	Tumor tissue	Methylation-specific PCR	Associated with clinical grade and metastasis. High sensitivity and specificity reveals its diagnostic potential (87–94%, AUC = 0.92).	Braga et al., 2018b
miR-150	Downregulated	Tumor tissue	Real time PCR	Correlated with shorter progression free survival	Wilczynski et al., 2018
miR-4443 and miR-5195-3p	Downregulated	Tumor tissue	Real time PCR	Correlates with metastasis and tumor progression	Ebrahimi and Reiisi, 2019
miR-148a	Downregulated	Plasma samples and ovarian cancer cell line	Real time PCR	Associated with poor prognosis, tumor growth and metastasis	Gong et al., 2016
(hsa-miR-135, 150,−340, 625, 1908, 3187,−96,−196b,−449c, and−1275)	Downregulated	Tumor tissue	Small RNA sequencing, quantitative RT-PCR	Associated with survival	Chen et al., 2018
miR-595	Downregulated	Tumor tissue	qRT-PCR	Correlated with shorter overall survival	Zhou et al., 2017
miR-498	Downregulated	Tumor tissue	qPCR	Correlated with shorter overall survival and progression free survival	Cong et al., 2015
miR-9	Downregulated	Tumor tissue and ovarian cancer cell line	Real time PCR, luciferase reporter assay, Western Blot, Methylation study, RNAi technique, and cytotoxicity Assay	Resistance to paclitaxel by targeting CCNG1.	Li X. et al., 2015
miR-508-3p	Downregulated	Tumor tissue and ovarian cancer cell line	System biology approach, qRT-PCR, methylation PCR, RNA sequencing, immuno blot	Predictor for mesenchymal subtype and metastasis	Zhao et al., 2019
hsa-miR-1273g-3p	Downregulated	Serum samples of recurrent epithelial ovarian cancer patients	Microarray and qRT-PCR	Prognostic biomarker for recurrence	Günel et al., 2018

Recently, Braga et al. described methylation of miR-9-1, miR-9-3, and miR-130b which strongly correlated with progression of OC (Braga et al., 2018a). Different histotype of ovarian carcinomas reflect differential expression of specific miRNAs which might serve as a valid biomarker. Agostini et al. reported significant overexpression of miR-192, miR-194, and miR-215 in mucinous subtype of ovarian carcinomas. However their expression was downregulated in other subtypes and sex cord-stromal tumors (Agostini et al., 2018).

A list of promising aberrantly expressed miRs which could be of prognostic and predictive relevance in ovarian cancer has been summarized in Table 11. A lower ratio of miR-221 to miR-222 significantly correlated with worse overall survival in predominantly high grade, advanced stage sporadic ovarian carcinomas (Wurz et al., 2010). Downregulation of miR-141, miR−200a, miR-200b, miR-200c, and miR-429 correlated with poor progression free survival. Moreover, multivariate analysis of relevant clinicopathological variables such as debulking status, stage and grade of tumor revealed the correlation of miR-429 expression with recurrence-free survival (Leskela et al., 2010). Downregulated miR-422b and miR-34c correlated with decreased disease-specific survival in HGSOC patients with BRCA1/2 abnormalities (Lee et al., 2009).

In ovarian cancer, overexpression of miR-214 has been specifically associated with the degradation of PTEN mRNA which further leads to the activation of Akt pathway and has been correlated with platinum resistance (Yang H. et al., 2008). Downregulation of miR-Let7i has been reported in platinum-resistance ovarian tumors; however its gain of function resulted in restoration of drug sensitivity of chemoresistance OC cells (Yang N. et al., 2008).

Several studies have recently highlighted the diagnostic and prognostic relevance of several miRNAs, their association with overall survival of patients and have shown that they could serve as putative biomarker as well as therapeutic target for ovarian cancer management. For instance, Li et al. have reported tumor suppressive role of miR-542-3p, which directly targets CDK14 and was observed significantly downregulated in EOC tissue and OC cell lines (Li et al., 2019).

Si et al. highlighting the therapeutic significance of miR-27a in OC, reported miR-27a mediated regulation of proliferation, chemosensitivity and invasion of OC by targeting Cullin 5 (CUL5) (Si et al., 2019, p. 5). Another study by Jia et al. reported the tumor suppressive role of miR-34 in regulation of tumor proliferation via inducting autophagy and apoptosis and suppression of cell invasion by targeting Notch 1 (Jia et al., 2019, p. 1). Wang et al. utilizing integrated meta-analysis approach have shown the oncogenic role of miRNA-27a by mediating FOXO1 and its inhibition could serve as a new strategy in combating ovarian cancer (Wang Z. et al., 2018, p. 1). Hu et al. identified miR-934 as an oncogene in OC by directly targeting BRMS1L, and thus could serve as a therapeutic marker (Hu et al., 2019). It has been reported that miR-1294 was identified to be downregulated in EOC and correlated with tumor progression and shorter overall survival, thereby could serve as an independent prognostic indicator (Guo W. et al., 2018).

Liu et al. provided insights into the oncogenic role of microRNA-96 (miR-96-5p) in ovarian cancer. Its significant overexpression was found in tissue as well as serum samples. Overexpression of miR-96-5p was correlated with increased proliferation and migration by suppressing Caveolae1 (CAV1) and inhibiting AKT signaling pathway and its downstream proteins (Cyclin D1 and P70), thus implying that miR-96-5p could serve as a promising therapeutic target for ovarian cancer (Liu et al., 2019, p. 1). Similarly, Chaluvally-Raghavan et al. reported that miR551b-3p which is an oncogenic microRNA, directly upregulates STAT3 expression and further deregulates proliferation and metastasis *in vivo* and *in vitro*. Reduced expression of STAT3 in OC cells *in vitro* and *in vivo* via anti-miR551b-3p leads to reduction in growth of ovarian tumor *in vivo*, thereby implying that it could serve as promising therapeutic target in future for ovarian cancer (Chaluvally-Raghavan et al., 2016).

In another study, miR-152 mediated suppression of tumor proliferation along with promotion of apoptosis via repression of ERBB3 was reported, thus demonstrating miR-152 as a potential therapeutic target (Li et al., 2017, p. 3). Liu et al. reported association of miR-506 with better response to therapy as well as long PFS and overall survival in OC patients. Further, it sensitized cancer cells to chemotherapy by directly targeting RAD51 and thus could be of therapeutic importance (Liu et al., 2015).

10 miRs which were identified using genome wide MicroRNA expression profiling were capable to discriminate malignant tissue samples from normal with a sensitivity of 97% and specificity of 92% (Wang et al., 2014). Biamonte et al. have reported tumor suppressive role of miR-let-7g and significant association of its reduced expression in both tissue as well as serum with chemoresistance in advanced stage EOC patient which reflects its potential as a predictive biomarker to monitor response to chemotherapy (Biamonte et al., 2019). Kobayashi et al. have shown significant overexpression of serum miR-1290 in advance stage HGSOC in comparison to early stage. Moreover, it was capable to discriminate patients with HGSOC from patients with malignancies of other histological subtypes with a sensitivity of 47% and specificity of 85% (AUC = 0.76), thus reflecting diagnostic potential of miR-1290 for HGSOC (Kobayashi et al., 2018).

Mahmoud et al. examined the diagnostic significance of serum miR-21 and reported that its upregulation was significantly negatively correlated with Programmed Cell Death-4 (PDCD4) expression in EOC patients (Mahmoud et al., 2018). Another study highlighted significantly elevated expression of serum exosomal miR-93, miR-145, and miR-200c in OC. Moreover, the sensitivity for miR-145 and miR-200c was 91.6 and 90.0% which was far superior in comparison with CA125, thus these serum exosomal microRNAs could be of diagnostic relevance for preoperative diagnosis of OC (Kim et al., 2019). miR-21 was observed significantly overexpressed in the sera of EOC patients and its elevated expression correlated with shorter overall survival (Xu et al., 2013). Further, downregulation of serum miR-25 and miR-93 and upregulation of miR-7 and miR-429 have been reported in OC patients. In addition, the sensitivity and specificity achieved by these four serum miRs were 93 and 92% to discriminate cancer patients from non-neoplastic control samples, deciphering their diagnostic significance in EOC. Moreover serum miR-429 correlated with overall survival and could serve as an independent prognostic indicator (Meng et al., 2015). Findings from another study reveal the relevance of serum miR-141 and miR-200c in OC diagnosis and prognosis. Both of these miRs were identified to be overexpressed in serum of EOC patients; however miR-200c displayed a descending expression trend across tumor stage (early to advance) while an escalating expression trend was observed in case of miR-141. Moreover, the sensitivity for miR-141 and miR-200c were 0.69 and 0.72 with a specificity of 0.72 and 0.70, respectively, to discriminate cancerous samples from normal control [AUC = 0.75 and 0.79, respectively]. Furthermore, high serum miR-200c correlated with higher survival rate. On contrary, low serum miR-141 correlated with higher survival rate (Gao and Wu, 2015).

Langhe et al. using Exiqon platform explored a 4-miR panel in serum of EOC patients for their diagnostic utility and found that these miRs were significantly downregulated in EOC patients. Furthermore these miRs target WNT signaling, AKT/mTOR signaling and TLR-4/MyD88 to regulate ovarian cancer progression and resistance (Langhe et al., 2015). Overexpression of serum miR-200a, miR-200b, and miR-200c which have been observed in EOC patients, correlates with aggressive disease progression and could be indicative of disease prognosis and patient survival (Zuberi et al., 2015). Higher serum concentration of exosomal miR-200b and miR-200c correlated with shorter overall survival, which suggests its prognostic relevance. (Meng et al., 2016b) Serum miR-200a, miR-200b, and miR-200c differentiated cancerous and benign tumors with 83% sensitivity and 100% specificity, which reflect that these miRs could be of diagnostic utility (Meng et al., 2016a).

These miRs though hold great potential for their utility in ovarian cancer management; however its therapeutical implementation still remains a challenge. To address this, well-designed clinical study as well as validated methodologies is essentially warranted.

#### Expert Commentary

It is now well-established that DNA methylation occurs very early in malignant transformation and their utility as biomarker holds great promise to overcome the false positive detection of ovarian cancer using current standard serum marker CA125. In this review, we highlight the recent epigenetic biomarkers analyzed in tissue and body fluids for early detection of OC. Strikingly; to date no single epigenetic biomarker facilitating early diagnosis of OC has made transition to the clinics. The probable reasons for this could be: the heterogeneous nature of EOC, difference in sample processing, assay design, technique used and approach could explain the variations observed in methylation frequencies amongst various studies for individual genes. Most of the studies for methylation analysis of genes were conducted on small sample size and in particular the normal control samples were insufficient to conclude the specificity of the assay. Therefore, further studies on larger sample size are necessary to be conducted to determine the potential of methylation if it could serve as biomarker for early EOC screening or not. Another limitation is the absence of standardized reference value for methylation analysis when trying to analyze if a particular locus is hyper or hypomethylated. To overcome this, currently, methylation cut off points which are based on already published reports or consensus are used.

The majority of the reports highlight the methylation status of gene or genes in a panel. No epigenetic biomarker screening study has been performed till date. However, for the detection figures approaching current screening modalities (89.5% sensitivity and 99.8% specificity) has been achieved by Ibanez de Caceres et al. (2004) with 82% sensitivity and 100% specificity (Ibanez de Caceres et al., 2004). All 30 control cases showed 0 false positive rate and further replication of the study on the basis of this sample size would give a false positive rate between 0 and 11.4% (95% confidence interval), thereby indicating that perfect specificity would unlikely hold up in the follow-up studies. In view of these considerations regarding the study of Ibanez de Caceres et al. are left to follow-up studies to shed light on. However, none of the report has been further validated undertaking follow up studies on a larger cohort and prospective study design thereby limiting the utility of the reported findings.

Molecular analysis of epigenetic modification (methylation) in circulating cell free tumor DNA in fluids serves as a novel, non-invasive approach for identification of potential promising cancer biomarkers, which can be performed at multiple time points and probably better reflects the prevailing molecular profile of cancer. Very few studies analyzing the methylation status of genes in blood-based assay for ovarian cancer diagnosis has been reported. Careful precision handling and processing of liquid biopsy for cell free DNA extraction is critically needed.

#### Future Prospects

Over the last decade, an exponential progress in DNA methylation based biomarker development has been witnessed. Owing to the stability of DNA and methylation pattern, a number of cfDNA as well as tissue based screening assay has paved its way into clinics. The commercial success of several tests based on DNA methylation biomarkers for early detection of colon, lung and prostate cancer and prediction of bladder cancer along with various markers under validation study shows that the time for transition into clinics can be relatively rapid. New technologies which allow rapid identification of methylation signatures directly from blood will facilitate sample-to answer solutions thereby enabling next-generation point of care molecular diagnostics. Moreover, ongoing work on liquid biopsies together with the recent advanced technologies such as digital PCR, bisulfite sequencing, methyl immune-precipitation coupled with next-generation sequencing, and methylation arrays along with advanced statistical data analysis may mitigate the problematic issues for the development of non-invasive method thereby overcoming the existing challenges to personalized medicine.

## Author Contributions

AS wrote the manuscript. SG and MS edited the final version of the manuscript.

### Conflict of Interest Statement

The authors declare that the research was conducted in the absence of any commercial or financial relationships that could be construed as a potential conflict of interest.
