# Supplementary figures and images for: Epigenetic Biomarkers in the Management of Ovarian Cancer: Current Prospectives
Source: Front Cell Dev Biol. 2019 Sep 19;7:182. doi: 10.3389/fcell.2019.00182 (PMC6761254; doi:10.3389/fcell.2019.00182)

## Supplementary material

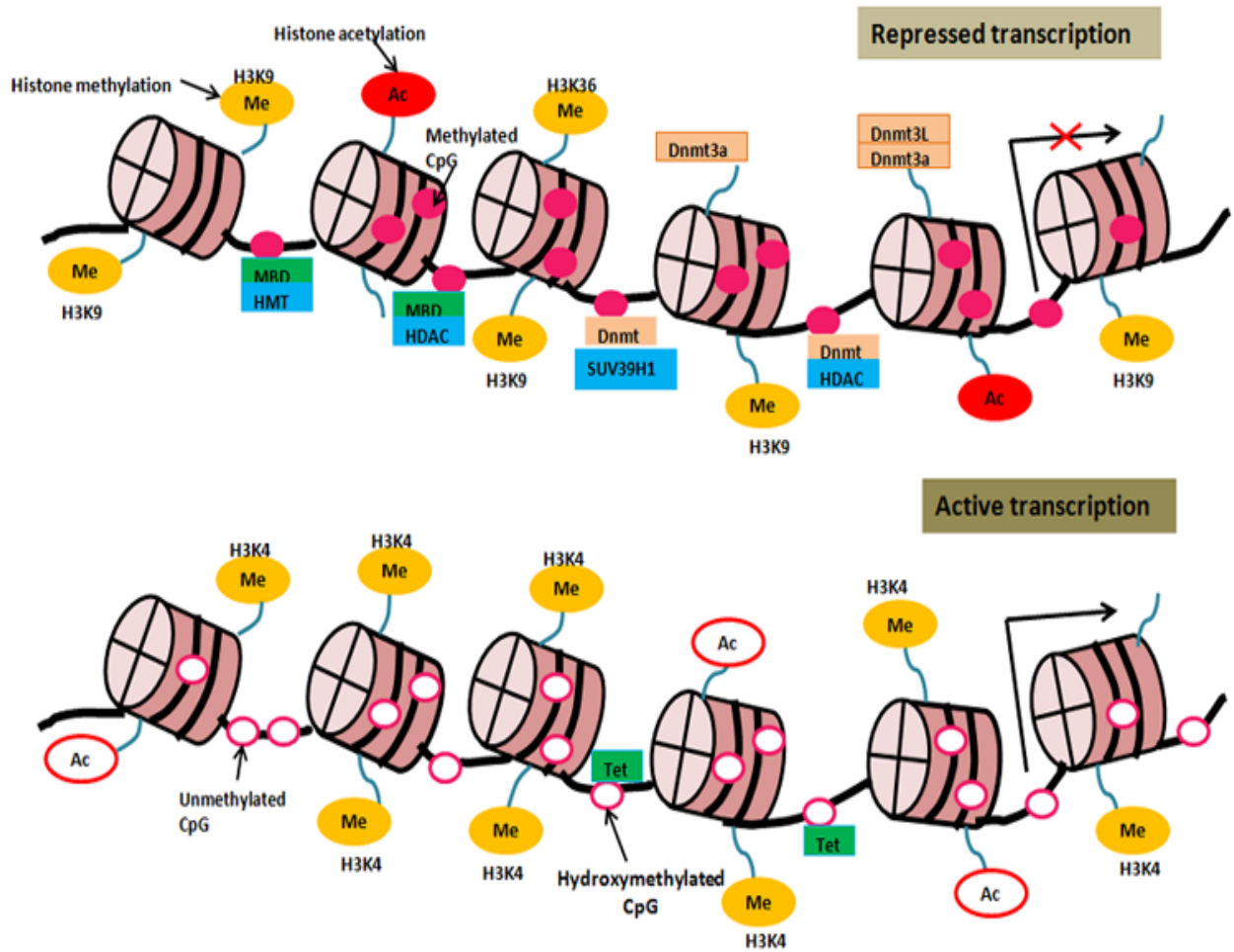

Supplementary Figure 1. Epigenetic regulation of gene expression

Supplement: Supplementary file 1 [file Data_Sheet_1.PDF]
